# Classical and Bayesian random-effects meta-analysis models with sample quality weights in gene expression studies

**DOI:** 10.1186/s12859-018-2491-9

**Published:** 2019-01-09

**Authors:** Uma Siangphoe, Kellie J. Archer, Nitai D. Mukhopadhyay

**Affiliations:** 1 Office of Biostatistics, Center for Drug Evaluation and Research, U.S. Food and Drug Administration, Silver Spring, Maryland USA; 2 Division of Biostatistics, College of Public Health, The Ohio State University, Columbus, Ohio USA; 3 Department of Biostatistics, Virginia Commonwealth University, Richmond, Virginia USA

**Keywords:** Random-effects model, Bayesian random-effects model, Meta-analysis, Study heterogeneity, Gene expression, Sample quality weights, Alzheimer’s disease

## Abstract

**Background:**

Random-effects (RE) models are commonly applied to account for heterogeneity in effect sizes in gene expression meta-analysis. The degree of heterogeneity may differ due to inconsistencies in sample quality. High heterogeneity can arise in meta-analyses containing poor quality samples. We applied sample-quality weights to adjust the study heterogeneity in the DerSimonian and Laird (DSL) and two-step DSL (DSLR2) RE models and the Bayesian random-effects (BRE) models with unweighted and weighted data, Gibbs and Metropolis-Hasting (MH) sampling algorithms, weighted common effect, and weighted between-study variance. We evaluated the performance of the models through simulations and illustrated application of the methods using Alzheimer’s gene expression datasets.

**Results:**

Sample quality adjusting within study variance (*w*_*P*6_) models provided an appropriate reduction of differentially expressed (DE) genes compared to other weighted functions in classical RE models. The BRE model with a uniform(0,1) prior was appropriate for detecting DE genes as compared to the models with other prior distributions. The precision of DE gene detection in the heterogeneous data was increased with the DSLR2*w*_*P*6_ weighted model compared to the DSL*w*_*P*6_ weighted model. Among the BRE weighted models, the *w*_*P*6_weighted- and unweighted-data models and both Gibbs- and MH-based models performed similarly. The *w*_*P*6_ weighted common-effect model performed similarly to the unweighted model in the homogeneous data, but performed worse in the heterogeneous data. The *w*_*P*6_weighted data were appropriate for detecting DE genes with high precision, while the *w*_*P*6_weighted between-study variance models were appropriate for detecting DE genes with high overall accuracy. Without the weight, when the number of genes in microarray increased, the DSLR2 performed stably, while the overall accuracy of the BRE model was reduced. When applying the weighted models in the Alzheimer’s gene expression data, the number of DE genes decreased in all metadata sets with the DSLR2*w*_*P*6_weighted and the *w*_*P*6_weighted between study variance models. Four hundred and forty-six DE genes identified by the *w*_*P*6_weighted between study variance model could be potentially down-regulated genes that may contribute to good classification of Alzheimer’s samples.

**Conclusions:**

The application of sample quality weights can increase precision and accuracy of the classical RE and BRE models; however, the performance of the models varied depending on data features, levels of sample quality, and adjustment of parameter estimates.

**Electronic supplementary material:**

The online version of this article (10.1186/s12859-018-2491-9) contains supplementary material, which is available to authorized users.

## Background

Although modern sequencing technologies such as ribonucleic acid sequencing and next-generation sequencing have been developed, microarrays have been a widely used high-throughput technology in gathering large amounts of genomic data [1, 2]. Due to small sample sizes in single microarray studies, microarray studies are combined with meta-analytic techniques to increase statistical power and generalizability of the results [1, 3].

Common meta-analysis techniques applied in gene expression studies included combining of *p*-values, rank values, and effect sizes. Examples of the p-value based methods include Fisher’s method, Stouffer’s method, minimum *p*-value method, maximum p-value method, and adaptively weighted Fisher’s method. The rank-based methods include *rt*h ordered *p*-value method, naïve sum of ranks, naïve product of ranks, rank product, and rank sum methods. The effect-size based methods include fixed-effects (FE) and random-effects (RE) models.

Appropriateness of the meta-analysis techniques in gene expression data depends on types of hypothesis testing: HSA, HSB, or HSC as described in [4–6]. Maximum p-value and naïve sum of rank methods were appropriate for HSA hypothesis that detected DE genes across all studies. The *rt*h ordered *p*-value method and two-step DerSimonian and Laird estimated RE models were appropriate for HSB hypothesis that detected DE genes in one or more studies. DerSimonian and Laird (DSL) and empirical Bayes estimated RE models, including our two-step estimated RE model using DSL and random coefficient of determination (*R*^*2*^) method were appropriate for HSC hypothesis that detected DE genes in a majority of combined studies [4–6].

Some of these methods may be limited in their application. The p-value based methods are limited in reporting summary effects and addressing study heterogeneity [3, 7–9]. The rank-based methods are robust towards outliers and applied without assuming a known distribution [8, 10]; however, their results are dependent on the influence of other genes included in microarrays [1]. The FE model assumes that total variation is derived from a true effect size and a measurement error [3]; however, the effect may vary across studies in real-world applications. Concurrently, although the RE model can address study-specific effects and accounts for both within and between study variation, the between study variation or the heterogeneity in effect sizes is unknown. Many frequentist-based methods have been developed to estimate the between study variation. More details can be found in [6, 9, 11, 12].

The RE models are commonly applied in gene expression meta-analysis. Classical RE models assume studies are independently and identically sampled from a population of studies. However, an infinite population of studies may not exist and studies may be designed based on results of previous studies, thus potentially violating an independence assumption. Bayesian random-effects (BRE) models have been used to allow for uncertainty of parameters. The uncertainty is expressed through a prior distribution and a summary of evidence provided by the data is expressed by the likelihood of the models. Multiplying the prior distribution and the likelihood function results in a posterior distribution of the parameters [13, 14].

Sample quality has substantial influence on results of gene expression studies [15, 16]. The degree of heterogeneity may differ due to inconsistencies in sample quality. Low heterogeneity can be found in meta-analyses containing good quality samples, while high heterogeneity arises in meta-analyses containing poor quality samples. In our recent study, we evaluated the relationships between DE and heterogeneous genes in meta-analyses of Alzheimer’s gene expression data. We detected some overlapped DE and heterogeneous genes in meta-analyses containing borderline quality samples, while no heterogeneous genes were detected in meta-analyses containing good quality samples [6]. Obviously, data obtained from borderline (poor) quality samples can increase study heterogeneity and reduce the efficiency of meta-analyses in detecting DE genes [17, 18].

In this study, we implemented a meta-analytic approach that includes sample-quality weights to take study heterogeneity into account in RE and BRE models. The gene expression data therefore would consist of up-weighted good quality samples and down-weighted borderline quality samples. Therefore in the Methods section we first review quality assessments of microarray samples, sample-quality weights, RE models, BRE models, weighted RE models, and weighted BRE models. We then describe our simulation studies and application data. Our results are then presented followed by discussion and conclusions.

## Methods

This section describes quality assessments of microarray samples, sample-quality weights, RE models, BRE models, weighted RE models, and weighted BRE models.

### Microarray quality assessments

Affymetrix GeneChips and Illumina BeadArrays have been widely used single channel microarrays. Quality assessments in Affymetrix arrays include the 3′:5′ ratios of two-control genes: beta-actin, and glyceraldehyde-3-phosphate dehydrogenase (GAPDH); the percent of number of genes called present; the array-specific scale factor; and the average background [15, 19]. A 3′:5′ ratio close to 1 indicates a good quality sample while a ratio > 3 suggests a poor quality sample, resulting from problems of RNA extraction, cDNA synthesis reaction, or conversion to cRNA [15, 20]. Additionally, the percent present calls should be consistent among all arrays hybridized and generally should range from 30 to 60% [21]. The scale factor is used to assess overall expression levels with an acceptable value within 3-fold of one another. The proportion of up- and down-regulated genes should be consistent at the average signal intensity so that the expression among arrays can be comparable. The average background should also be consistent across all arrays [15]. For Illumina BeadArrays, quality assessments include the average and standard deviation of intensities, the detection rate, and the distance of specific probe intensities to the overall mean intensities of all samples [22–24].

### Random-effects models

In this section, we provided a brief summary of the random-effects models implemented in this study. The hypothesis settings for detecting DE genes in meta-analysis of gene expression data are described in the supplemental material.

#### DerSimonian-Laird model (DSL)

An unbiased standardized mean difference in expression between groups (*y*_*ig*_) can be obtained for each gene *g* as described in Hedges et.al. (1985) and Choi et.al. (2003) as:

1 $$ {y}_{ig}={y}_{ig}^{\prime }-\frac{3{y}_{ig}^{\prime }}{4\left({n}_{ig}-2\right)-1},\kern0.5em {y}_{ig}^{\prime }=\frac{{\overline{x}}_{ig(a)}-{\overline{x}}_{ig(c)}}{{\mathrm{s}}_{ig}}, $$

2 $$ {s}_{ig}^2=\frac{\left({n}_{ig(a)}-1\right){s}_{ig(a)}^2+\left({n}_{ig(c)}-1\right){s}_{ig(c)}^2}{n_{ig(a)}+{n}_{ig(c)}-2}, $$

where ${\overline{x}}_{ig(a)}$and ${\overline{x}}_{ig(c)}$ represent the mean expression of case (a) and control (c) groups in *i*th study, *i = 1,…,k*; s_*ig*_and *n*_*ig*_are an estimate of the pooled standard deviation across groups and the total sample size in the *i*th study; and*y*_*ig*_is obtained as the correction for sample size bias. The estimated variance of *y*_*ig*_ is ${\sigma}_{ig}^2=\left({n}_{ig(a)}^{-1}+{n}_{ig\left(\mathrm{c}\right)}^{-1}\right)+{y}_{ig}^2{\left(2\left({n}_{ig(a)}+{n}_{ig(c)}\right)\right)}^{-1}$. The model of effect-size combination is based on a two-level hierarchical model:

3 $$ {\displaystyle \begin{array}{c}{y}_{ig}={\theta}_{ig}+{\varepsilon}_{ig},\kern1.25em {\varepsilon}_{ig}\sim N\left(0,{\sigma}_{ig}^2\right)\\ {}{\theta}_{ig}={\beta}_g+{\delta}_{ig},\kern1em {\delta}_{ig}\sim N\left(0,{\tau}_g^2\right),\end{array}} $$

where *y*_*ig*_ is the effect for gene *g* in *i*th study, *i = 1,…,k*; *θ*_*ig*_ is the true difference in mean expression; ${\sigma}_{ig}^2$is the within-study variability representing sampling errors conditional on the *i*th study; *β*_*g *_is the common effects or average measure of differential expression across datasets for each gene or the parameter of interest; *δ*_*ig*_is the random effect; and ${\tau}_g^2$ is the between-study variability. The RE model is defined when there is between-study variation [11, 25]. The estimator for ${\tau}_g^2$is typically obtained using DerSimonian-Laird (DSL) estimator [26, 27] as

4 $$ {\widehat{\tau}}_{DSL(g)}^2=\max \left\{0,\frac{Q_g-\left({k}_g-1\right)}{S_{1g}-\left({S}_{2g}/{S}_{1g}\right)}\right\}, $$

where ${Q}_g={\sum}_{i=1}^k{w}_{ig}{\left({y}_{ig}-{\widehat{\beta}}_g\right)}^2,\kern0.5em {w}_{ig}={\sigma}_{ig}^{-2},\kern0.5em {\widehat{\beta}}_g=\frac{\sum_{i=1}^k{w}_{ig}{y}_{ig}}{\sum_{i=1}^k{w}_{ig}},\kern0.5em {S}_{rg}={\sum}_{i=1}^k{w}_{ig}^r$, and *r* = {1, 2}. For each gene, we estimated ${\widehat{\beta}}_g\left({\widehat{\tau}}_{DSL(g)}^2\right)$ with ${w}_{ig}={\left({\sigma}_{ig}^2+{\widehat{\tau}}_{DSL(g)}^2\right)}^{-1}$ using a generalized least squares method to obtain statistics *z*_*DSL*(*g*)_. More details can be found in [11, 25].

#### Two-step estimate model (DSLR2)

The ${\widehat{\tau}}_{DSLR2(g)}^2$was estimated by the DSL method in the first step and iterated with random-effect coefficients of determination (${R}_{DSL(g)}^2$) in the second step. In other words, we assumed ${\delta}_{ig}\sim N\left(0,{R}_{DSL(g)}^2\right)$ and replaced ${\widehat{\tau}}_{DSL(g)}^2$ by ${R}_{DSL(g)}^2$ in the second-step estimation. ${\widehat{\tau}}_{\mathrm{DSL}(g)}^2$ and ${R}_{\mathrm{DSL}(g)}^2$ are a function of *τ*^2^(**Y**_*g*_ − **β**_*g*_), so its bias does not influence the unbiasedness of the treatment and random effects [6, 12]. The ${\widehat{\tau}}_{DSLR2(g)}^2$on the zero-to-one scale provides a lower minimum sum of squared error (MSSE) than the ${\widehat{\tau}}_{DSL(g)}^2$ estimate. The ${R}_{DSL(g)}^2$ measuring the strength of study heterogeneity can also be used to compare variation of genes in different meta-analyses to decide which studies should be included in the meta-analysis [28]. The estimates of treatment effects, its variance, z-statistics, and random effects are obtained as

5 $$ {\widehat{\beta}}_g\left({R}_{DSL(g)}^2\right)=\frac{\sum_{i=1}^k{\left({\sigma}_{ig}^2+{R}_{DSL(g)}^2\right)}^{-1}{y}_{ig}}{\sum_{i=1}^k{\left({\sigma}_{ig}^2+{R}_{DSL(g)}^2\right)}^{-1}}, $$

6 $$ Var\left[{\widehat{\beta}}_g\left({R}_{DSL(g)}^2\right)\right]=\frac{1}{\sum_{i=1}^k{\left({\sigma}_{ig}^2+{R}_{DSL(g)}^2\right)}^{-1}}, $$

7 $$ {z}_g\left({R}_{DSL(g)}^2\right)=\frac{{\widehat{\beta}}_g\left({R}_{DSL(g)}^2\right)}{\sqrt{Var\left({\widehat{\beta}}_g\left({R}_{DSL(g)}^2\right)\right)}}\kern0.5em \sim N\left(0,1\right), $$

8 $$ {\widehat{\delta}}_{ig}\left({R}_{DSL(g)}^2\right)=\frac{R_{DSL(g)}^2}{\sigma_{ig}^2+{R}_{DSL(g)}^2}\left({y}_{ig}-{\widehat{\beta}}_g\left({R}_{DSL(g)}^2\right)\right) $$

When compared to the DSL method, the DSLR2 method had a relatively better sensitivity and accuracy in detecting DE genes under HSC hypothesis testing and a higher precision when the proportion of truly DE genes in the metadata was higher [6]. The DSLR2 method performed well with a low computational cost and almost all significantly DE genes identified were genes among the significantly DE genes identified using the DSL method. However, similar to the DSL method, the performance of the DSLR2 method can be reduced when sample sizes in single studies are restricted (e.g., < 60 in both arms) and the normality assumption of the meta-analysis outcome does not hold [6].

The RE models may be inefficient due to improper distributional assumptions. A permutation technique that is not based on a parametric distribution was applied to assess statistical significance of the common effect [11]. A modified BH method was used to control the FDR for multiple testing in the RE models [29]. We obtained the modified FDR by the order statistics of the actual and permuted z-statistics *z*_(*g*)_ = (*z*_(1)_ ≤ ⋯ ≤ *z*_(*G*)_) and ${z}_{(g)}^r=\left({z}_{(1)}^r\le \cdots \le {z}_{(G)}^r\right)$ as

9 $$ {FDR}_g=\frac{\left(1/R\right){\sum}_{r=1}^R{\sum}_{(g)=1}^GI\left(|{z}_{(g)}^r|\ge {z}_{\alpha}\right)}{\sum_{\left(\mathrm{g}\right)=1}^GI\left(|{z}_{(g)}|\ge {z}_{\alpha}\right)}, $$

where *α* is the significance threshold of the single test, *g* is an index of genes *1,…,G*, and *r* is an index of permutation *1,…,R.*

#### Bayesian random-effects model (BRE)

The BRE models are different from the classical RE model in that the data and model parameters in the BRE models are considered to be random quantities [30]. The BRE models were used to allow for the uncertainty of the between-study variance in this study. The model for gene *g* is given by

$$ {y}_{ig}\left|\ {\theta}_{ig}\right.\sim N\left({\theta}_{ig},{\sigma}_{ig}^2\right), $$

$$ {\theta}_{ig}\left|\ {\beta}_g,{\tau}_g\right.\sim N\left({\beta}_g,{\tau}_g^2\right), $$

$$ {\beta}_g\sim N\left(0,1000\right), $$

10 $$ {\tau}_g\sim uniform\left(a,b\right) andgamma\left(\alpha, \beta \right). $$

The kernel of the posterior distribution can be written as

11 $$ {\displaystyle \begin{array}{l}p\left({\beta}_g,{\theta}_{1g},\dots, {\theta}_{kg},{\tau}_g^2\right)\propto p\left({\boldsymbol{\theta}}_g|{\mathbf{y}}_g,{\upsigma}_g^2\right)p\left({\beta}_g,{\tau}_g^2|{\boldsymbol{\theta}}_g\right)\\ {}\\ {}\kern8em \propto {\prod}_{i=1}^kp\left({\theta}_{ig}|{y}_{ig},{\sigma}_{ig}^2\right)p\left({\theta}_{ig}|{\beta}_g,{\tau}_g^2\right)\pi \left({\beta}_g\right)\pi \left({\tau}_g^2\right),\end{array}} $$

where ${\mathbf{y}}_g=\left({y}_{1g},\dots, {y}_{kg}\right),{\boldsymbol{\upsigma}}_g^2=\left({\sigma}_{1g}^2,\dots, {\sigma}_{kg}^2\right)$, and ***θ***_*g*_ = (*θ*_1*g*_, …, *θ*_*kg*_) for gene *g* in the *i*th study; *i = 1,…,k.* The *π*(*β*_*g*_) and $\pi \left({\tau}_g^2\right)$ are non-informative priors given as *β*_*g*_ ∼ *N*(0, 1000), and*τ*_*g*_∼uniform (a,b) and gamma (α,β).

The choice of prior distributions for scale parameters can affect analysis results, particularly in small samples. With scale parameters, the distributional form and the location of the prior distributions are decided [31]. Uniform distributions are appropriate non-informative priors for ${\tau}_g^2$ [13]. We conducted simulations to select appropriate priors for ${\tau}_g^2$, allowing the maximum (b) of the uniform distribution to be b∈{0.005, 0.001, 0.05, 0.01, 0.5, 0.1, 1, 5, 10} and b~Gamma(1,2). The potential choices of the appropriate priors were selected based on parameters obtained from an Alzheimer’s gene expression data [6] in order to further apply the results.

### Sample-quality weights

The quality control (QC) criteria indicative of poor quality samples we used were the 3′:5’ GAPDH ratio > 3 and/or percent of present calls < 30% for Affymetrix arrays; and detection rate < 30% for Illumina BeadArrays, in addition to data visualizations [15, 20]. Poor quality samples were excluded before data preprocessing. Theoretically, an optimal weight for meta-analysis is the inverse of the within-study variance. The variance of weighted mean (${\widehat{\beta}}_g$) is minimized when the individual weights are taken from the variance of the samples *y*_*ig*_. A high variance therefore gives low weights in meta-analysis [32, 33]. In this study, the weights corresponding to the QC indicators fall into two categories: standardized ratio weights and zero-to-one weights (Table 1).

**Table 1:** List of sample quality weights

Standardized ratio weights (*w*_*S*, *ij*_)	Zero-to-one weights (*w*_*P*, *ij*_)	
${w}_{S1}={\left({\sigma}_g^2+{s}_{ij}{\widehat{\tau}}_g^2\right)}^{-1}$ ${w}_{S2}={\left({s}_{ij}{\sigma}_{ig}^2+{\widehat{\tau}}_g^2\right)}^{-1}$ ${w}_{S3}={\left({s}_{ij}\left({\sigma}_{ig}^2+{\widehat{\tau}}_g^2\right)\right)}^{-1}$ ${w}_{S4}={2}^{-\left({\sigma}_{ig}^2+{s}_{ij}{\widehat{\tau}}_g^2\right)}$ ${w}_{S5}={2}^{-\left({s}_{ij}{\sigma}_{ig}^2+{\widehat{\tau}}_g^2\right)}$ ${w}_{S6}={2}^{-\left({s}_{ij}\left({\sigma}_{ig}^2+{\widehat{\tau}}_g^2\right)\right)}$	${w}_{P1}\in \left\{{2}^{-{s}_{ij}},0.01{\overset{\sim }{p}}_{ij}\right\}$ ${w}_{P2}={\left({\sigma}_{ig}^2+\left(1-{w}_{P1}\right){\widehat{\tau}}_g^2\right)}^{-1}$ ${w}_{P3}={\left(\left(1-{w}_{P1}\right){\sigma}_{ig}^2+{\widehat{\tau}}_g^2\right)}^{-1}$ ${w}_{P4}={\left(\left(1-{w}_{P1}\right)\left({\sigma}_{ig}^2+{\widehat{\tau}}_g^2\right)\right)}^{-1}$ ${w}_{P5}={\left({\sigma}_{ig}^2+{\widehat{\tau}}_g^{2\left({w}_{P1}\right)}\right)}^{-1}$ ${w}_{P6}={\left({\sigma}_{ig}^{2\left({w}_{P1}\right)}+{\hat{\tau}}_g^2\right)}^{-1}$ ${w}_{P7}={\left({\left({\sigma}_{ig}^2+{\widehat{\tau}}_g^2\right)}^{\left({w}_{P1}\right)}\right)}^{-1}$	${w}_{P8}={2}^{-\left({\sigma}_{ig}^2+\left(1-{w}_{P1}\right){\widehat{\tau}}_g^2\right)}$ ${w}_{P9}={2}^{-\left(\left(1-{w}_{P1}\right){\sigma}_{ig}^2+{\widehat{\tau}}_g^2\right)}$ ${w}_{P10}={2}^{-\left(\left(1-{w}_{P1}\right)\left({\sigma}_{ig}^2+{\widehat{\tau}}_g^2\right)\right)}$ ${w}_{P11}={2}^{-\left({\sigma}_{ig}^2+{\widehat{\tau}}_g^{2\left({w}_{P1}\right)}\right)}$ ${W}_{P12}={2}^{-\left({\sigma}_{ig}^{2\left({w}_{P1}\right)}+{\widehat{\tau}}_g^2\right)}$ ${w}_{P13}={2}^{-\left(\left({\sigma}_{ig}^2+{\widehat{\tau}}_g^2\right){w}_{P1}\right)}$

#### Standardized ratio weights (*w*_*S*,*ij*_)

12 $$ {S}_{ij}=\left|\frac{R_{ij}-1}{SD\left({R}_i\right)}\right|\kern0.75em \in \kern0.5em \left(0,\infty \right), $$

$$ {w}_{S, ij}=f\left({S}_{ij},{\sigma}_i^2,{\tau}^2\right), $$

where *R*_*ij*_ is a quality indicator, i.e. 3′:5’ GAPDH ratio of the *j*th sample in the *i*th study, *SD*(*R*_*i*_)is the standard deviation of the quality indicator in the *i*th study, *w*_*S*1 − *S*3_ ∈ (0, ∞), and *w*_*S*4 − *S*8_ ∈ (0, 1). *f*(.) is a function of sample-quality weights with the within and between study variances as shown in Table 1. A low value of the *S*_*ij*_ indicates good quality samples, providing high values of standardized ratio weights (*w*_*S*,*ij*_) to give more weight on the expression data.

#### Zero-to-one weights (*w*_*P*,*ij*_)

13 $$ {P}_{ij}=\left\{\begin{array}{c}\ {\tilde{P}}_{ij}(0.01)\ \\ {}{2}^{-{S}_{ij}}\ \end{array}\right\}\kern0.75em \in \kern0.5em \left[0.01,\dots, 1.0\right], $$

$$ {w}_{P, ij}=f\left({P}_{ij},{\sigma}_i^2,{\tau}^2\right), $$

where ${\tilde{P}}_{ij}$ and *S*_*ij*_ is the percent of present calls and the standardized quality indicators of the *j*th sample in the *i*th study, respectively, *w*_*P*1 − *P*7_  ∈ (0, ∞), and *w*_*P*8 − *P*13_  ∈ (0, 1). A high value of the *P*_*ij*_ weights indicate good quality samples, providing high values of zero-to-one weights (*w*_*P*,*ij*_) to give more weight on the expression data.

The weights are primarily selected based on availability of quality indicators, such as 3′:5’ GAPDH ratio in Affymetrix arrays or detection rate in Affymetrix arrays and Illumina BeadArrays. Both the 3′:5’ GAPDH ratio and detection rate can be converted to the zero-to-one weights via *w*_*P*1_.

### Weighted random-effects models

An appropriate weight was chosen based on the precision and accuracy of the DSL weighted and DSLR2 weighted models in detecting DE genes via simulations and were used to weight the expression data and to adjust the common effect and the between-study variance in the BRE model.

#### Weighted DSL and DSLR2 models

The log_2_ normalized intensity data were weighted with an appropriate weight obtained from the DSL and DSLR2 weighted models. The weighted mean $\left({\overline{x}}_{ig(a)}\right)$ and weighted sample variance $\left({\mathrm{s}}_{ig(a)}^2\right)$ of the normalized intensity data in each group were calculated:

14 $$ {\overline{x}}_{ig(a)}={\sum}_{j=1}^{n_{ig\left(\mathrm{a}\right)}}{w}_{ijg(a)}{x}_{ijg(a)}/{\sum}_{j=1}^{n_{ig\left(\mathrm{a}\right)}}{w}_{ijg(a)}, $$

15 $$ {\mathrm{s}}_{ig(a)}^2=\frac{\sum_{j=1}^{n_{ig\left(\mathrm{a}\right)}}{w}_{ijg(a)}{\left({x}_{ijg(a)}-{\overline{x}}_{ig(a)}\right)}^2}{S_{1g(a)}-\left({S}_{2g(a)}/{S}_{1g(a)}\right)};\kern0.5em {S}_{rg(a)}={\sum}_{j=1}^{n_{ig\left(\mathrm{a}\right)}}{w}_{ijg(a)}^r,\kern0.5em r=\left\{1,2\right\}, $$

*x*_*ijg*(*a*)_ is the log_2_ normalized intensity data for gene *g* of the *j*th sample in the case (a) group and in the *i*th study, *n*_*ig*(a)_ is the sample size of case (a) group for gene *g* in the *i*th study, and *w*_*ijg*(*a*)_ is the sample-quality weight of the *j*th sample in the case (a) group in the *i*th study for the gene *g*. The same calculations were applied for the weighted mean $\left({\overline{x}}_{ig(c)}\right)$ and the weighted sample variance $\left({\mathrm{s}}_{ig(c)}^2\right)$ in the control (c) group. The unbiased standardized mean difference of the expression between groups were re-calculated and re-combined using the DSL and DSLR2 models (Eq.1 and Eq.2).

#### Weighted common effect model

We adjusted the common effect in the BRE model (Eq.10) by multiplying with an average weight over the total sample in the *i*th study for gene *g*$\left({\overline{w}}_{ig}={\sum}_{j=1}^{n_{ig(a)}+{n}_{ig\left(\mathrm{c}\right)}}{w}_{ijg}/\left({n}_{ig(a)}+{n}_{ig\left(\mathrm{c}\right)}\right)\right)$. The BRE weighted common effect model for gene *g* is given by

$$ {y}_{ig}\mid {\theta}_{ig}\operatorname{}\kern0.5em \sim N\left({\theta}_{ig},{\sigma}_{ig}^2\right), $$

$$ {\theta}_{ig}\mid {\beta}_g{\overline{w}}_{ig},{\tau}_g\operatorname{}\sim N\left({\beta}_g{\overline{w}}_{ig},{\tau}_g^2\right), $$

$$ {\beta}_g\sim N\left(0,1000\right), $$

16 $$ {\tau}_g\sim \mathrm{uniform}\left(\mathrm{a},\mathrm{b}\right)\ \mathrm{and}\ \mathrm{gamma}\left(\upalpha, \upbeta \right) $$

#### Weighted between-study variance model

We adjusted the between-study variance in the BRE model (Eq.10) by multiplying with an average weight over the total sample in the *i*th study for gene *g*$\left({\overline{w}}_{ig}={\sum}_{j=1}^{n_{ig(a)}+{n}_{ig\left(\mathrm{c}\right)}}{w}_{ijg}/\left({n}_{ig(a)}+{n}_{ig\left(\mathrm{c}\right)}\right)\right)$. The BRE weighted between-study variance model for gene *g* is given by

$$ {y}_{ig}\mid {\theta}_{ig}\operatorname{}\kern0.5em \sim N\left({\theta}_{ig},{\sigma}_{ig}^2\right), $$

$$ {\theta}_{ig}\mid {\beta}_g,{\tau}_g{\overline{w}}_{ig}\operatorname{}\sim N\left({\beta}_g,{\tau}_g^2{\overline{w}}_{ig}\right), $$

$$ {\beta}_g\sim N\left(0,1000\right), $$

17 $$ {\tau}_g\sim \mathrm{uniform}\left(\mathrm{a},\mathrm{b}\right)\ \mathrm{and}\ \mathrm{gamma}\left(\upalpha, \upbeta \right) $$

Example WinBUGS code appears in the supplemental material.

The weighted common effect and the weighted between study variance in the BRE models with a uniform(0,1) prior were implemented in both unweighted and weighted data using Gibbs and Metropolis-Hasting (MH) sampling algorithms [14, 34]. Two chains each with 20,000 iterations, a 15,000 burn-in period, and a thinning of 3 was performed for all Bayesian models. The convergence of the models was assessed using the Gelman and Rubin diagnostic [34]. Since the posterior distribution was normal and symmetric, the posterior mean was standardized by posterior standard deviation. A Benjamini and Hochberg (BH) procedure was applied to control the false discovery rate (FDR) for multiple gene testing, so that the BRE and classical RE models could be compared throughout the study. Seven BRE models for unweighted and weighted data, Gibbs and MH sampling algorithms, weighted common effect, and weighted between-study variance were implemented as shown in Table 2.

**Table 2:** Bayesian random-effects (BRE) models by data features, sampling algorithms, and weighted inference models

	BRE Models						
	1	2	3	4	5	6	7
Data features							
Unweighted normalized intensity data	✓	✓	✓				
Weighted normalized intensity data				✓	✓	✓	✓
Sampling algorithms							
Gibbs sampling	✓	✓	✓	✓	✓	✓	
Metropolis-Hasting sampling							✓
Weighted inference models							
Unweighted model	✓			✓			✓
Weighted common effect		✓			✓		
Weighted between-study variance			✓			✓	

The DE genes were defined as those with FDR less than 5%. Unsupervised hierarchical clustering using Ward’s method and one minus Pearson’s correlation coefficient for measures of similarities were used to graphically present the DE genes in the individual analysis of Alzheimer’s gene expression data using a heatmap.

### Simulation setting

Simulated datasets were generated using an algorithm described in previous studies [4–6]. A brief summary of the algorithm is as follows:

1. Five studies each with 2000 genes were generated (800 clustering and 1200 non-clustering genes). The clustering genes with the same correlation pattern within their clusters were equally allocated into 40 clusters.
2. Gene expression levels among clustering and non-clustering genes were assumed to follow a multivariate normal distribution ${\left({X}_{gc{1}^{\prime }},\dots, {X}_{gc{40}^{\prime }}\right)}^T\sim MVN\left(0,{\Sigma}_{ck}\right),$ 1 ≤ *k* ≤ 5, 1 ≤ *c* ≤ 40, ${\sum}_{ck^{\prime }}\sim {W}^{-1}\left(\psi, 60\right),$ and *ψ* = 0.5*I*_20 × 20_ + 0.5*J*_20 × 20_, and a standard normal distribution, respectively.
3. Truly DE genes were generated with uniform(0.5,3), accounted for 10% of the total genes, and equally classified into 5 groups (*t*_*g*_ = 1, …, 5). On average each group included 200 true genes. As the RE models appropriated under HSC, 120 genes in more than 50% of the combined studies were defined as the truly DE genes.
4. Truly heterogeneous genes constituted 15% of the total genes, implied by the random effects with uniform(0.5,3), and proportionally allocated into truly DE and not truly DE gene groups. The heterogeneous gene was defined by a significant random effect, where the gene expression was not identical across studies.
5. Sample-quality weights were assumed to follow beta distributions(*α* = 10, *β* = 1) for the zero-to-one weights and normal distributions  *N*(0, 0.6) for the standardized ratio weights.

The N, G, K, and H denote the number of samples, the number of genes, the number of studies, the number of studies containing heterogeneous genes, respectively, all of which varied in different simulations. Because the simulation results under the same algorithms on 2000 and 10,000 genes were similar [6] and implementing Bayesian models requires intensive computations, we conducted the simulations on 2000 genes. Eight simulated metadata sets: two sets for the weighted and unweighted methods in the homogeneous data (H0), and each two of six sets for the weighted and unweighted methods in the heterogeneous data (H1, H2, and H3) were generated. A thousand simulations each with 1000 permutations of group labels were implemented for all DSL and DSLR models, and without permutation for the BRE models with different uniform(0,b) priors; b∈{0.005, 0.001, 0.05, 0.01, 0.5, 0.1, 1, 5, 10, and 100}, and b~Gamma(1,2) prior.

### Evaluations of methods in simulations

Because RE models were suitable under HSC hypothesis: detecting DE genes in a majority of combined studies [5, 6], the models were anticipated to detect DE genes in more than 50% of combined studies, *r = 3* for meta-analysis of five studies. We evaluated the number of detected DE genes, minimum sum squared error (MSSE), precision, accuracy, and area under receiver operating characteristic curve (AUC). Precision was calculated as the proportion of truly DE genes correctly identified as significant over the total number of genes declared significant. Accuracy was calculated as the proportion of genes correctly identified as being truly DE genes or not truly DE genes over the total of evaluated genes. The accuracy of the tests was also determined using AUC, where AUC ∈ (0.5, 0.7],  AUC ∈ (0.7, 0.9] and AUC ∈ (0.9, 1.0] represent low, moderate, and high accuracy, respectively [35, 36]. All statistical methods and simulations were implemented using programs and modified programs from *limma, metafor, GeneMeta, MAMA, Rjags, R2jags, Coda* in the R programming environment.

Four publicly available Alzheimer’s disease (AD) gene expression datasets of post-mortem hippocampus brain samples were applied: GSE1297 [37], GSE5281 [38], GSE29378 [39], and GSE48350 [40]. After data preprocessing, quantile normalization, and data aggregating [20, 41–44], our meta-analysis was performed on 12,037 target genes in 131 subjects (68 AD cases and 63 controls). We examined the strength of study heterogeneity by considering five ways of metadata sets as previously described in [6] and defined in the caption of Figs. 5 and 6. The metadata A, B, D, E may contain heterogeneous data due to a relatively high *R*^2^, while the metadata C had a relatively low *R*^2^or contained homogenous data. The 3′:5’ GAPDH ratio was used as a quality indicator in this analysis. The 3′:5’ GAPDH ratio was converted to the zero-to-one weight, *w*_*P*6_, via *w*_*P*1_.

## Results

**Fig. 1:**
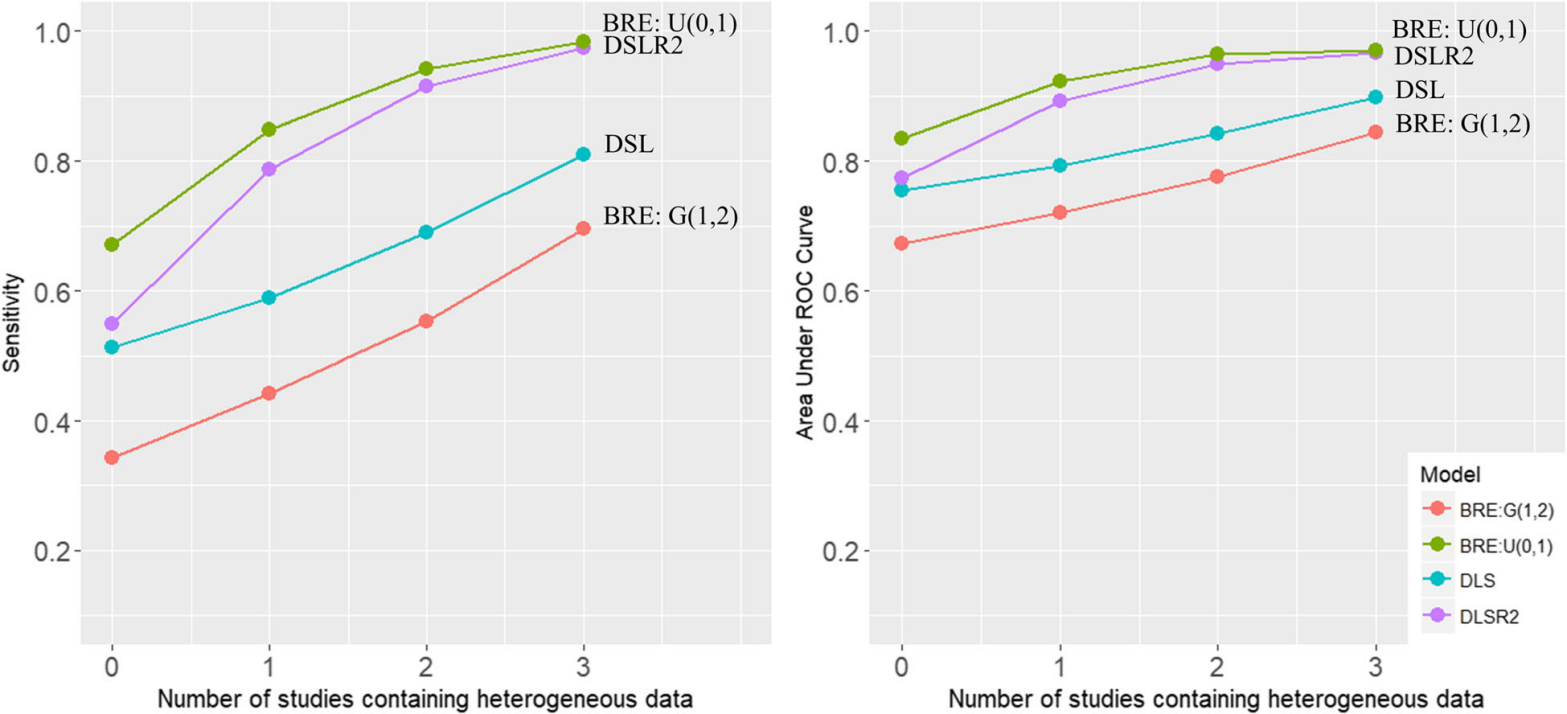
Sensitivity and area under ROC curve of the random-effects models with Dersimonian-Laird (DSL), two-step (DSLR2), and Bayesian random-effects models (BRE) with uniform(0,1) and gamma(1,2) priors for between-study variance under the HSC hypothesis testing. H0, H1, H2, and H3 are the number of {0, 1, 2, and 3} studies containing heterogeneous genes. H0 represents homogenous data. The number of truly DE genes in the simulated data was 120 genes

Table 3 presents the performance of the DSL and DSLR2 models, and the BRE models with different prior distributions. All of the BRE models converged with the potential scale reduction factor close to 1. The BRE model with a uniform(0,1) prior detected more DE genes than the DSL and DSLR2 models. The BRE model with a uniform(0,b) prior where b = {0.001, 0.01, 0.1, 0.005, 0.05, 0.5} detected too many DE genes, particularly in the heterogeneous data, while the BRE model with a uniform(0,5), uniform(0,10), uniform(0,100), and gamma(1,2) prior detected too few DE genes. The DSLR2 model had the lowest MSSE, while the DSL model and the BRE model with a uniform(0,1) prior had similar MSSEs (Additional file 1: Figure S1). In addition, the DSL, DSLR2, BRE with a uniform(0,1) prior detected DE genes with high precision in the homogeneous data, moderate precision in the heterogeneous data, and high accuracy in all datasets. The DSLR2 and BRE with a uniform(0,1) prior had a higher AUC than the DSL model in the heterogeneous data (Fig. 1).

**Table 3:** Performance of random-effects models applied in simulated data

Model	Prior	No. DE Genes				MSSE				Precision				Accuracy				AUC			
		H0	H1	H2	H3	H0	H1	H2	H3	H0	H1	H2	H3	H0	H1	H2	H3	H0	H1	H2	H3
DSL	–	65	74	92	124	2.9	2.9	2.9	2.9	0.95	0.95	0.91	0.79	0.97	0.97	0.98	0.98	0.76	0.79	0.84	0.90
DSLR2	–	69	104	139	198	1.7	1.7	1.7	1.7	0.95	0.91	0.79	0.59	0.97	0.98	0.98	0.96	0.77	0.89	0.95	0.97
BRE	U(0,0.001)	126	157	254	305	18.1	25.8	33.0	39.9	0.82	0.70	0.45	0.39	0.98	0.97	0.93	0.91	0.93	0.94	0.94	0.94
BRE	U(0,0.01)	218	324	404	436	10.5	16.0	20.0	22.3	0.55	0.37	0.30	0.28	0.95	0.90	0.86	0.84	0.97	0.95	0.92	0.92
BRE	U(0,0.1)	181	269	354	391	9.4	14.3	17.8	19.8	0.66	0.45	0.34	0.31	0.97	0.93	0.88	0.86	0.98	0.96	0.94	0.93
BRE	U(0,1)	80	108	141	203	1.7	2.2	2.4	2.6	1.00	0.94	0.80	0.58	0.98	0.99	0.98	0.96	0.84	0.92	0.96	0.97
BRE	U(0,10)	11	9	9	12	1.0	1.1	1.1	1.1	1.00	1.00	1.00	0.96	0.95	0.94	0.94	0.95	0.54	0.54	0.54	0.55
BRE	U(0,100)	10	8	8	11	1.0	1.0	1.0	1.0	1.00	1.00	1.00	0.96	0.94	0.94	0.94	0.94	0.54	0.53	0.53	0.54
BRE	U(0,0.005)	329	447	520	546	10.6	16.1	20.1	22.4	0.37	0.27	0.23	0.22	0.90	0.84	0.80	0.79	0.94	0.91	0.89	0.89
BRE	U(0,0.05)	184	275	359	395	10.3	15.7	19.6	21.8	0.65	0.44	0.33	0.30	0.97	0.92	0.88	0.86	0.98	0.96	0.94	0.93
BRE	U(0,0.5)	137	167	253	330	3.0	4.4	5.3	5.7	0.86	0.71	0.47	0.36	0.99	0.98	0.93	0.89	0.98	0.98	0.96	0.94
BRE	U(0,5)	13	11	12	17	1.1	1.1	1.1	1.1	1.00	1.00	1.00	0.97	0.95	0.95	0.95	0.95	0.55	0.54	0.55	0.57
BRE	G(1,2)	41	53	69	94	1.7	2.0	2.1	2.1	1.00	1.00	0.97	0.89	0.96	0.97	0.97	0.98	0.67	0.72	0.78	0.84

DE: differentially expressed, MSSE: minimum sum of squared error, AUC: area-under ROC curve, DSL: Dersimonian-Laird model, DSLR2: two-step estimate of Dersimonian-Laird model, BRE: Bayesian random-effects model, U: uniform, and G: gamma. H0, H1, H2, and H3 are the number of {0, 1, 2, and 3} studies containing heterogeneous genes. H0 represents homogenous data. The number of truly DE genes in the simulated data was 120 genes under HSC hypothesis testing

Therefore, the DSLR2 and BRE models with a uniform(0,1) prior were appropriate for detecting DE genes in terms of an appropriate number of DE genes, a lower MSSE, a higher precision, and a higher AUC, particularly in the heterogeneous data. The BRE model with a uniform(0,1) prior particularly performed better than the DSLR2 model in the homogeneous data but performed similarly in the heterogeneous data.

### Weighted DSL and DSLR2 models

**Fig. 2:**
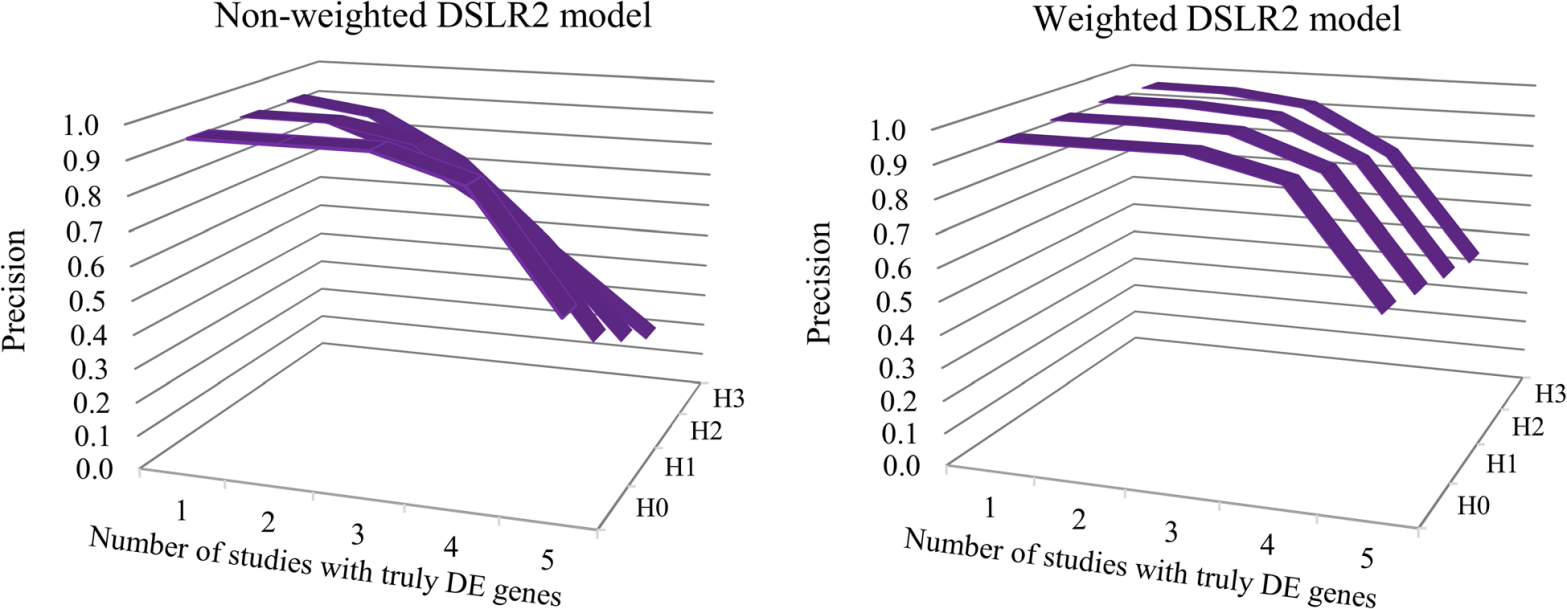
Precision of two-step random-effects models (DSLR2) with and without the proper weighted function: ${w}_{P6}={\left({\sigma}_{ig}^{2\left({w}_{P1}\right)}+{\widehat{\tau}}_g^2\right)}^{-1}$), ${w}_{P1}\in \left\{{2}^{-{S}_{ij}},0.01{\tilde{P}}_{ij}\right\}$, ${\tilde{P}}_{ij}$ denoted percent of present calls, *S*_*ij*_ denoted standardized quality indicators of the *j*th sample in the *i*th study. H0, H1, H2, and H3 are the number of {0, 1, 2, and 3} studies containing heterogeneous genes. H0 represents homogenous data. The number of truly DE genes in the simulated data was 120 genes under HSC hypothesis testing. DE: differentially expressed

With simulation results, the *w*_*P*6_ function was most appropriate for detecting DE genes in the DSL and DSLR2 models. The QC indicators adjusted the within study variance in the weighted function as:

18 $$ {w}_{P6}={\left({\sigma}_{ig}^{2\left({w}_{P1}\right)}+{\widehat{\tau}}_g^2\right)}^{-1}, $$

where ${w}_{P1}\in \left\{{2}^{-{S}_{ij}},0.01{\tilde{P}}_{ij}\right\}$, ${\tilde{P}}_{ij}$ denoted percent of present calls, *S*_*ij*_ denoted standardized quality indicators of the *j*th sample in the *i*th study. Fig. 2 presents the precision of the DSLR2 model with and without the *w*_*P*6_ function under different hypotheses in the homogeneous and heterogeneous data. The precision was increased with the DSLR2 weighted model in the heterogeneous data. The *w*_*P*6_ model provided an appropriate reduction of detected DE genes and MSSEs and higher precision as compared to the other weighted functions (Additional file 1: Tables S1 and S2). Similar results were found under different levels of sample quality (results not shown). The DSLR2 *w*_*P*6_ weighted model had a lower MSSE and detected more DE genes than the DSL *w*_*P*6_ weighted model in the heterogeneous data.

### Weighted Bayesian random-effects models

Table 4 presents the performance of the DSL*w*_*P*6_ and DSLR2*w*_*P*6_ models, and BRE weighted models. A uniform(0,1) prior for between study variance was applied in all BRE models. The BRE weighted Models 1, 3, 4, 6, and 7 in Table 4 detected more DE genes with a higher AUC than the DSL*w*_*P*6_ and DSLR2*w*_*P*6_ models. The *w*_*P*6_ weighted-data models performed similarly to the unweighted-data models (Models 2 vs. 5 and 3 vs. 6). The *w*_*P*6_weighted common-effect model performed similarly to the unweighted model in the homogeneous data, but performed worse in the heterogeneous data (Models 1 vs. 2). Additionally, the Gibbs- and MH-based models performed similarly on the *w*_*P*6_weighted-data model. The numbers of detected DE genes were reduced close to the number of truly DE genes and the precisions were increased while maintaining a high accuracy as compared to the performance in the unweighted-data Gibbs-based model (Models 4 and 7 vs. 1). For homogeneous and heterogeneous data, the Gibbs- and MH-based models with the *w*_*P*6_weighted-data performed similarly and were most appropriate for detecting DE genes with high precision (Models 4 and 7). The *w*_*P*6_weighted between-study variance models were most appropriate for detecting DE genes with high overall accuracy (Models 3 and 6).

**Table 4:** Performance of weighted random-effects models applied in simulated data

Model	No. DE Genes				MSSE				Precision				Accuracy				AUC			
	H0	H1	H2	H3	H0	H1	H2	H3	H0	H1	H2	H3	H0	H1	H2	H3	H0	H1	H2	H3
DSL*w*_*P*6_	62	62	64	65	2.9	3.0	3.0	3.0	0.95	0.96	0.96	0.96	0.97	0.97	0.97	0.97	0.75	0.75	0.75	0.76
DSLR2*w*_*P*6_	66	72	78	85	1.6	1.6	1.6	1.6	0.96	0.95	0.94	0.92	0.97	0.97	0.97	0.98	0.76	0.78	0.80	0.82
BRE with a uniform(0,1) prior																				
Model 1: Unweighted data, Gibbs	81	109	140	204	1.7	2.1	2.4	2.6	1.00	0.94	0.81	0.58	0.98	0.99	0.98	0.96	0.84	0.92	0.96	0.97
Model 2: Unweighted data, Gibbs, $\beta {\overline{w}}_{P6}$	81	66	51	39	6.0	6.2	6.5	6.9	1.00	1.00	0.97	0.93	0.98	0.97	0.96	0.96	0.84	0.77	0.71	0.65
Model 3: Unweighted data, Gibbs, ${\tau}^2{\overline{w}}_{P6}$	161	157	151	142	0.8	1.5	2.1	2.7	0.74	0.76	0.77	0.79	0.98	0.98	0.98	0.98	0.99	0.99	0.97	0.96
Model 4: Weighted data, Gibbs	81	87	92	100	1.8	2.2	2.7	3.1	1.00	0.99	0.97	0.93	0.98	0.98	0.98	0.98	0.84	0.86	0.87	0.89
Model 5: Weighted data, Gibbs, $\beta {\overline{w}}_{P6}$	81	65	51	39	6.3	6.5	6. 9	7.3	1.00	1.00	0.97	0.93	0.98	0.97	0.96	0.96	0.84	0.77	0.70	0.65
Model 6: Weighted data, Gibbs, ${\tau}^2{\overline{w}}_{P6}$	162	157	151	142	1.6	2.6	3.6	4.5	0.74	0.76	0.77	0.79	0.98	0.98	0.98	0.98	0.99	0.99	0.97	0.96
Model 7: Weighted data, MH	81	87	93	102	2.2	2.7	3.1	3.5	1.00	0.98	0.97	0.92	0.98	0.98	0.98	0.98	0.84	0.86	0.87	0.89

${\overline{w}}_{P6}$ is an average of *w*_*P*6_, ${w}_{P6}={\left({\sigma}_{ig}^{2\left({w}_{P1}\right)}+{\widehat{\tau}}_g^2\right)}^{-1}$over the total samples; ${w}_{P1}\in \left\{{2}^{-{S}_{ij}},0.01{\tilde{P}}_{ij}\right\}$, ${\tilde{P}}_{ij}$ denoted percent of present calls, *S*_*ij*_ denoted standardized quality indicators of the *j*th sample in the *i*th study. DE: differentially expressed, MSSE: minimum sum of squared error, AUC: area-under ROC curve, DSL: DerSimonian-Laird model, DSLR2: two-step estimate of DerSimonian-Laird model, BRE: Bayesian random-effects model, U: uniform, G: gamma, MH: Metropolis–Hastings algorithm. H0, H1, H2, and H3 are the number of {0, 1, 2, and 3} studies containing heterogeneous genes. H0 represents homogenous data. The number of truly DE genes in the simulated data was 120 genes under HSC hypothesis testing.

### Additional simulation results

Simulations with varying sample size, number of genes, and different levels of sample quality were conducted and some results were presented in the supplemental material. It is noteworthy that the BRE models identified less genes for sample sizes < 60. The DE gene detection and the MSSE were stable for sample sizes > 60. Specifically, the BRE with a U(0,1) had consistently high precisions and was able to maintain overall accuracies for all sample sizes > 60 (Additional file 1: Table S3). As anticipated, these findings were similar to the findings in the classical RE models [6]. When the number of genes in the analyses increased, the classical RE models performed stably, while the overall accuracy in the BRE model with a uniform(0,1) prior was reduced (Additional file 1: Table S4). For different levels of sample quality, the weights with higher sample quality detected more DE genes and had higher overall accuracy than the weights with lower sample quality (Additional file 1: Table S5).

### Application in Alzheimer’s gene expression data

**Fig. 3:**
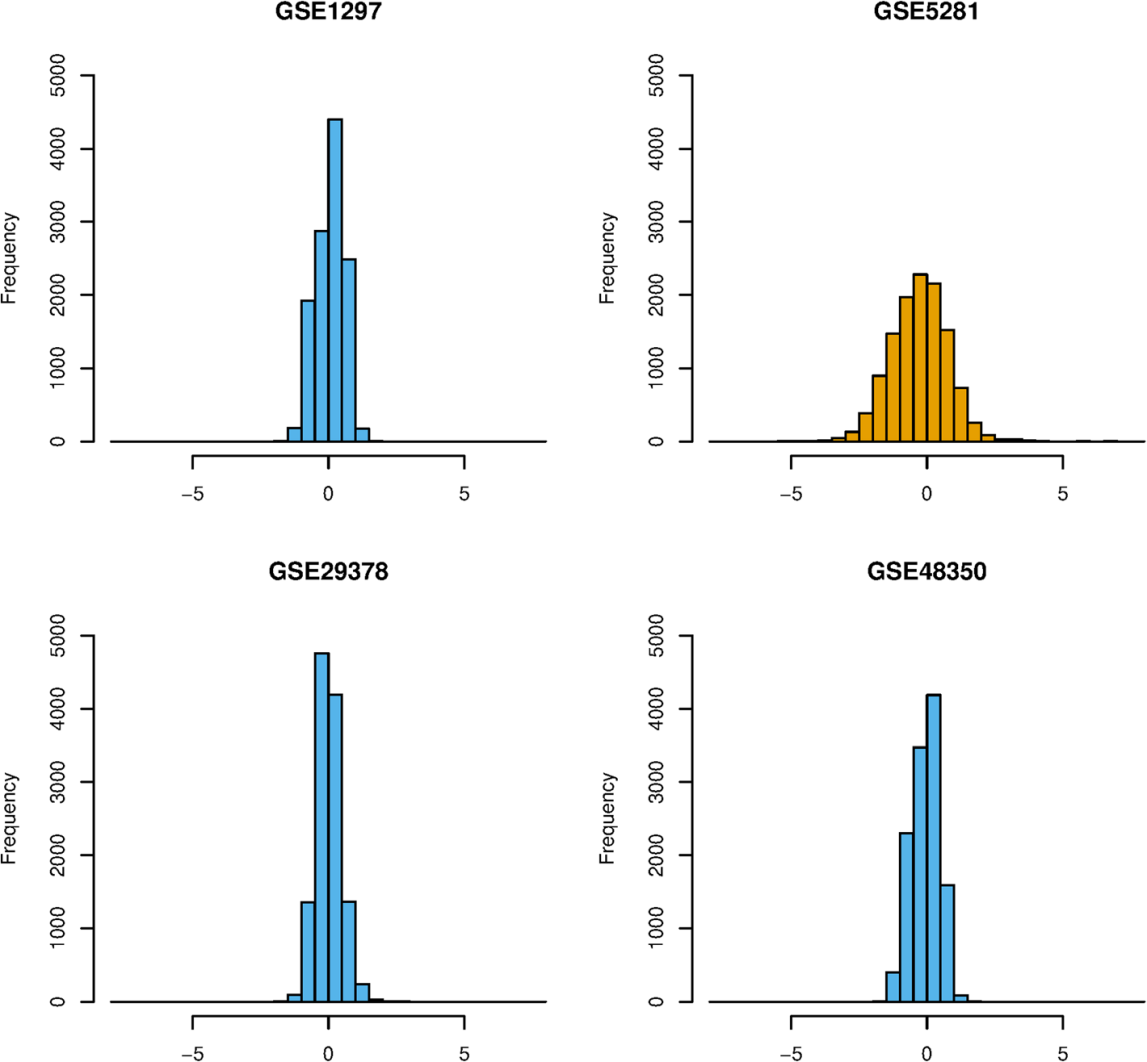
Distribution of unbiased standardized mean difference of gene expression (x-axis) between Alzheimer’s and control groups in GSE1297, GSE5281, GSE29378, and GSE48350 datasets

**Fig. 4:**
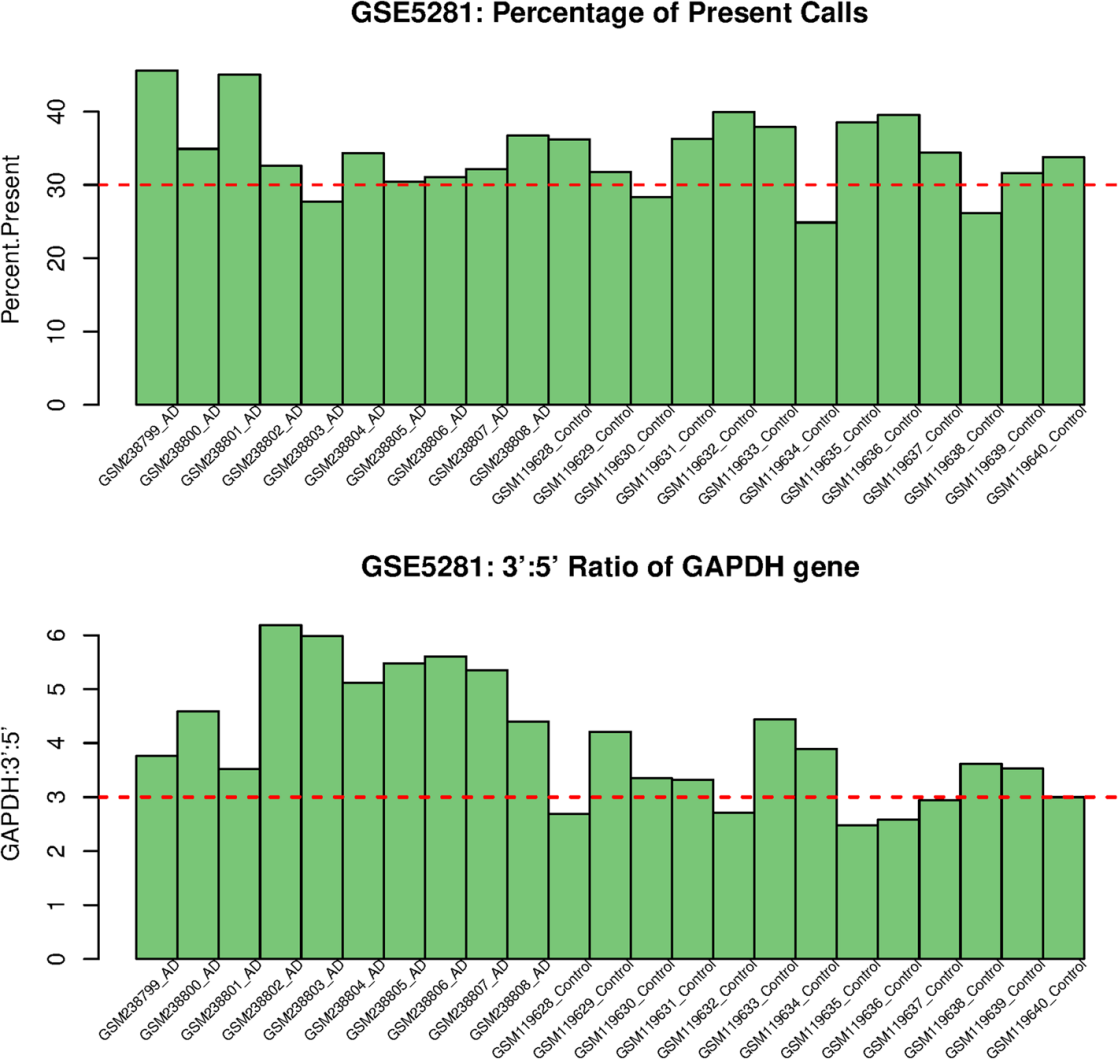
Percentage of present calls and 3′:5’ GAPDH ratio of GSE5281 samples

Our meta-analysis in the Alzheimer’s gene expression datasets was performed on 12,037 target genes in 131 subjects (68 AD cases and 63 controls). We primarily examined the strength of study heterogeneity by considering five ways of metadata sets as described in [6]. The metadata A, B, D, E may contain heterogeneous data due to a relatively high *R*^2^, while the metadata C had a relatively low *R*^2^or contained homogenous data. Figure 3 presents distribution of unbiased standardized mean differences of gene expression in the GSE5281 dataset, different from the other datasets. Figure 4 presents the percent of present calls and the 3′:5’ GAPDH ratio of the heterogeneous dataset.

**Fig. 5:**
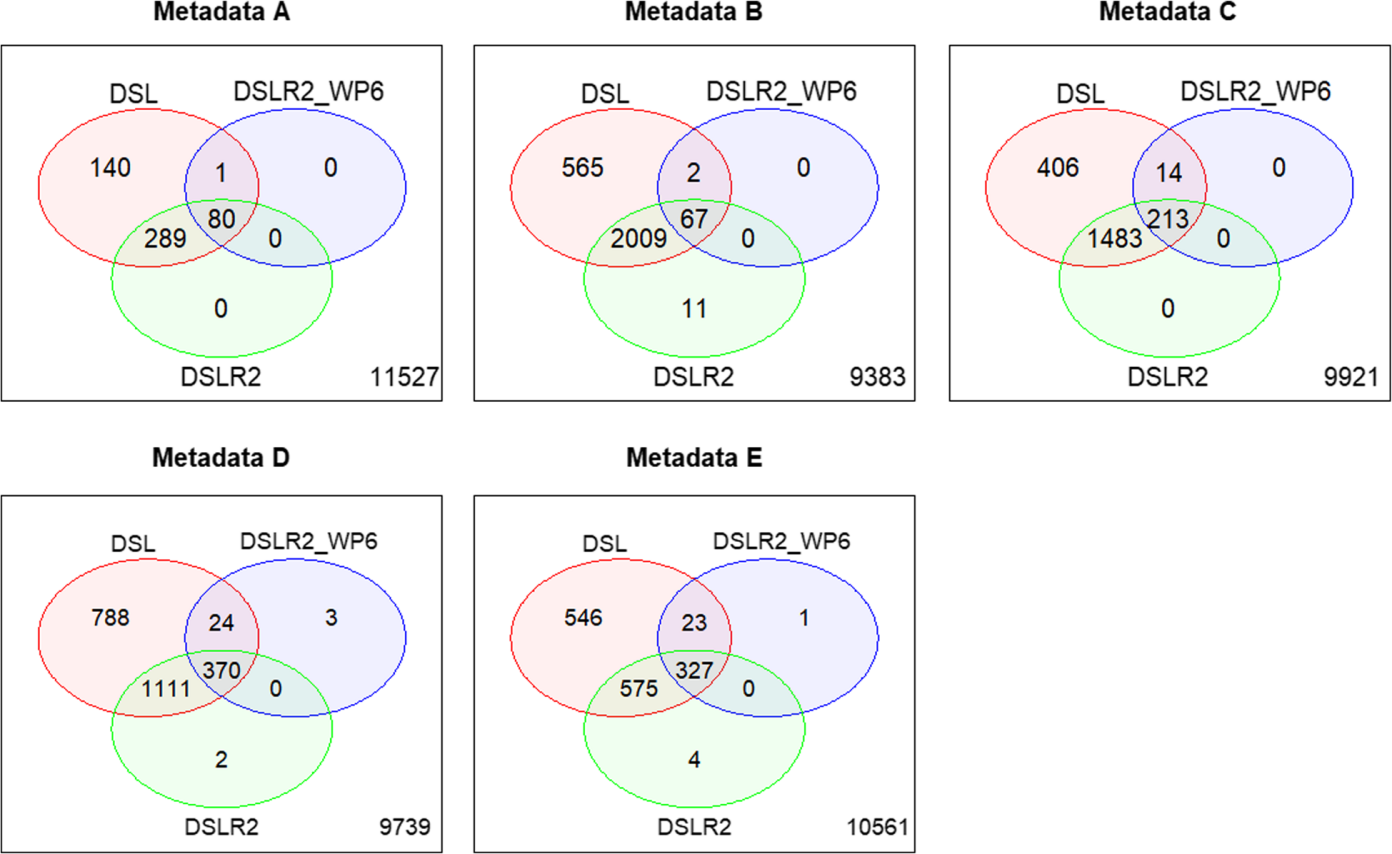
Venn diagrams present number of differentially expressed genes in Alzheimer's disease as compared to controls in white matter region using classical random-effects models: Dersimonian-Laird (DSL), two-step estimated (DSLR2) random-effects models and with the proper weighted function: ${w}_{p6}={\left({\sigma}_{ig}^{2\left({w}_{p1}\right)}+{\widehat{\tau}}_g^2\right)}^{-1}$ (DSLR2*w*_*P6*_), where ${w}_{p1}\in \left\{{s}^{-{S}_{ij}},0.01{\tilde{P}}_{ij}\right\}$, ${\tilde{P}}_{ij}$ denoted percent of present calls, *S*_*ij*_ denoted standardized quality indicators of the *j*th sample in the *i*th study. Metadata A: GSE1297, GSE5281, and GSE29378; B: GSE1297, GSE5281, and GSE48350; C: GSE1297, GSE29378, and GSE48350; D: GSE1297, GSE5281, GSE29378, and GSE48350; and E: GSE5281, GSE29378, and GSE48350

**Fig. 6:**
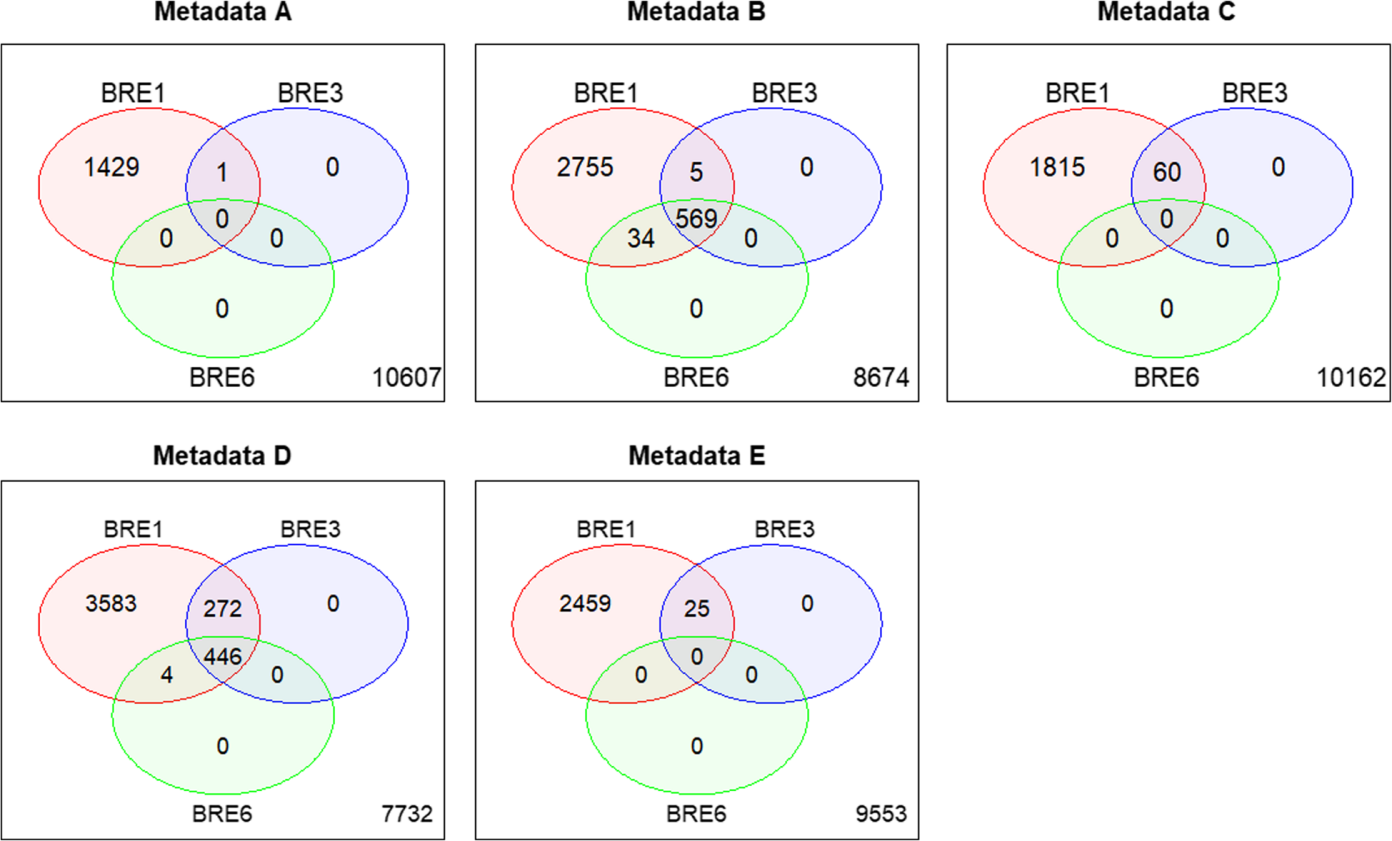
Venn diagrams present number of differentially expressed (DE) genes in Alzheimer's disease as compared to controls in white matter region using weighted Bayesian random-effects models - Model 1: unweighted BRE with uniform (0,1) model (BRE1), Model 3: unweighted data with Gibb sampling and *w*_*P6*_ weighted between study variance model (BRE3), Model 6: *w*_*P6*_ weighted data with Gibb sampling and *w*_*P6*_ weighted between study variance model (BRE6). This weighted function was applied: ${w}_{p6}={\left({\sigma}_{ig}^{2\left({w}_{p1}\right)}+{\widehat{\tau}}_g^2\right)}^{-1},{w}_{p1}\in \left\{{2}^{-{S}_{ij}},0.01{\tilde{P}}_{ij}\right\}$, ${\tilde{P}}_{ij}$ denoted percent of present calls, *S*_*ij*_ denoted standardized quality indicators of the *j*th sample in the *i*th study. Metadata A: GSE1297, GSE5281, and GSE29378; B: GSE1297, GSE5281, and GSE48350; C: GSE1297, GSE29378, and GSE48350; D: GSE1297, GSE5281, GSE29378, and GSE48350; and E: GSE5281, GSE29378, and GSE48350

Using the DSLR2*w*_*P*6_weighted model, the number of DE genes decreased in all metadata sets. Almost all the DE genes identified by the weighted model were genes among the significant DE genes identified by the unweighted DSL and DSLR2 models. The DE genes identified using the weighted model in the metadata C concurrently detected approximately 13% of the unweighted DSL and DSLR2 models (266/2116 genes and 213/1696 genes), respectively (Fig. 5). Likewise, the number of DE genes decreases with the *w*_*P*6_weighted between study variance (Models 3 and 6). Those DE genes were genes among the significant DE genes identified by the unweighted model (Model 1). Sixty and 446 DE genes were detected across the three weighted BRE models in the metadata C and D, respectively (Fig. 6). Among the unweighted or weighted classical RE and BRE models, 446 genes could potentially be down-regulated genes that may contribute to good classification of Alzheimer’s samples. Additional file 1: Figure S2 presents potential down-regulations of those genes in Alzheimer’s samples in each microarray dataset. Of note, no genes were detected using the weighted common-effect models (Models 2 and 5) and the weighted-data model (Models 4 and 7).

The lists of 213 and 446 DE genes can be found in Additional file 1: Tables S6 and S7, respectively, where 98 were the same genes. The identified DE genes participate in significant pathways such as cytoskeleton organization, actin filament bundle organization, synaptic transmission, regulation of biological quality, neutral lipid biosynthetic process, acylglycerol biosynthetic process, intermediate filament-based process, negative regulation of neuron projection development, cell-cell signaling, glutamate decarboxylation to succinate, stress fiber assembly, single-organism behavior, single-organism behavior, response to ethanol, cellular component assembly, neuron projection development, learning and long-term memory.

## Discussion

This study presents the performance of the classical RE and BRE models in meta-analysis of gene expression studies. We found the BRE model with a uniform(0,1) prior was appropriate for detecting DE genes as compared to the models with other prior distributions. The BRE model with a uniform(0,1) prior performed better than the DSLR2 model in the homogeneous data, but performed similarly in the heterogeneous data in terms of an appropriate number of detected DE genes, lower MSSE, higher precision, and higher AUC.

This is the first study to reveal an application of sample-quality weights to adjust the study heterogeneity in the classical RE and BRE models in microarray gene expression studies. The DSL and DSLR2 weighted models were implemented for the classical RE models. The unweighted and weighted data, Gibbs and MH sampling algorithms, weighted common effect, and weighted between-study variance were applied for the BRE models. We evaluated the performance of the models through simulation studies and through application to Alzheimer’s gene expression datasets.

With simulation results, the sample quality indicators adjusting the within study variance (*w*_*P*6_) in the classical RE models provided an appropriate reduction of detected DE genes and MSSEs, and higher precision as compared to the other weighted functions. The precision in detecting DE genes was increased with the DSLR2 *w*_*P*6_ weighted model in the heterogeneous data. The DSLR2 *w*_*P*6_ weighted model had a lower MSSE and detected more DE genes than the DSL *w*_*P*6_ weighted model in the heterogeneous data. Among the BRE weighted models, the *w*_*P*6_weighted- and unweighted-data models and both Gibbs- and MH-based models performed similarly. The *w*_*P*6_ weighted common-effect model performed similarly to the unweighted model in the homogeneous data, but performed worse in the heterogeneous data. The *w*_*P*6_weighted-data were appropriate for detecting DE genes with high precision, while the *w*_*P*6_weighted between-study variance models were appropriate for detecting DE genes with high overall accuracy.

The sample quality has substantial influence on results of gene expression studies [15]. Because variation of sample quality limited meta-analysis techniques to properly detect DE genes [45, 46] and the classical RE and BRE models allow flexibility in calculating *y*_*ig*_and its variance ${\sigma}_{ig}^2$ as well as study-specific adjustments [47], we developed approaches to up-weight good quality samples and down-weight borderline quality samples in the models. This compromised approach utilizes sample-quality information in the meta-analysis of microarray studies in detecting DE genes. The results in this study would benefit microarray gene expression studies because a large amount of microarray data are available in public repositories and unfortunately the data quality are often overlooked. However, the performance of the proposed models depends on not only degree of sample quality but also the number of studies, the number of genes, and sample sizes in the individual studies. The methods for controlling FDR under multiple testing would be another important aspect influencing gene expression results. Further intensive investigation of the topics would be the subject of future research.

The BRE models have the ability to allow for uncertainty of the parameter estimates in the model. Because the classical RE models tended to estimate ${\tau}_g^2$ as being zero, the variance of ${\widehat{\beta}}_g$were underestimated. The BRE models, in contrast, used the marginal posterior distribution of ${\tau}_g^2$ for ${\widehat{\beta}}_g$estimation, which do not depend on the point estimate of ${\tau}_g^2$. The BRE models can in turn increase the fitness of the models [48]. To illustrate, the precision was increased in the BRE*w*_*P*6_weighted-data models and the accuracy was increased in the BRE*w*_*P*6_weighted between-study variance models as compared to the classical RE weighted models. The BRE weighted models could be strengthened further in future research with informative priors using prior knowledge and historical information.

In real-world applications, BRE modeling in gene expression meta-analysis may be computationally intensive. To illustrate, a Gibbs-based model requires approximately 6 h per 10,000 gene set under supercomputers. A MH-based model requires twice longer than a Gibbs-based model. The computational time for a BRE model is highly dependent on not only types of the model, but also computer capacity. Computation time can indeed be another concern for model selection.

## Conclusions

This study applies sample-quality weights to adjust the study heterogeneity in the random-effects meta-analysis models. This meta-analytic approach can increase precision and accuracy of the classical and Bayesian random-effects models in gene expression meta-analysis. However, the performance of the weighted models varied depending on data feature, levels of sample quality, and adjustment of parameter estimates.

## Additional file

Additional file 1: **Table S1.** Number of differentially expressed (DE), minimum sum of squared errors (MSSE), precision, and accuracy of non-weighted and weighted random-effects models with Dersimonian-Laird (DSL) estimate applied in simulated data. **Table S2.** Number of differentially expressed (DE), Minimum sum of squared errors (MSSE), precision, and accuracy of non-weighted and weighted random effects meta-analysis model with two-step Dersimonian-Laird (DSLR2) estimate applied in simulated data. **Figure S1.** Number of differentially expressed genes and minimum sum of squared errors of Dersimonian-Laird (DSL), two-step (DSLR2)‚ and Bayesian random-effects (BRE) models with different lengths of uniform priors for between-study variance estimation in simulated data. **Table S3.** Performance of Bayesian random-effects models by different levels of sample sizes (some results from homogenous simulated datasets). **Table S4.** Performance of classical and Bayesian random-effects models by different numbers of genes (some results from H1 heterogeneous simulated datasets). **Table S5.** Performance of weighted random-effects models applied with two levels of sample-quality weights (some simulation results). **Figure S2.** Heatmaps of expression patterns of 446 differentially expressed genes in white matter in Alzheimer’s and control samples. The DE genes were detected across the three Bayesian meta-analysis models as shown in metadata D in Fig. 6. **Table S6.** List of 213 significantly differentially expressed genes in Alzheimer’s gene expression dataset. The DE genes detected across the DSLR2 *w*_*P*6_ weighted and DSLR2 and DSL unweighted models as shown in metadata C in Fig. 5. **Table S7.** List of 446 significantly differentially expressed genes in Alzheimer’s gene expression datasets. The DE genes detected across three Bayesian random-effect models (Models 1, 3, and 6) as shown in metadata D in Fig. 6. (PDF 886 kb)

## Abbreviations

AUC: Area under receiver operating characteristic curve
BH: Benjamini and Hochberg
BRE: Bayesian random-effects model
DE: Differentially expressed
DSL: DerSimonian and Laird
DSLR2: Two-step DerSimonian and Laird estimate
FDR: False discovery rate
FE: Fixed-effects
GAPDH: Glyceraldehyde-3-phosphate dehydrogenase
MH: Metropolis Hasting
MSSE: Minimum sum of squared error
QC: Quality control
RE: Random-effects
SD: Standard deviation
