# Supplementary material for: Classical and Bayesian random-effects meta-analysis models with sample quality weights in gene expression studies
Source: BMC Bioinformatics. 2019 Jan 9;20:18. doi: 10.1186/s12859-018-2491-9 (PMC6327440; doi:10.1186/s12859-018-2491-9)
Supplement: Supplementary file 1 — Table S1. Number of differentially expressed (DE), minimum sum of squared errors (MSSE), precision, and accuracy of non-weighted and weighted random-effects models with Dersimonian-Laird (DSL) estimate applied in simulated data. Table S2. Number of differentially expressed (DE), Minimum sum of squared errors (MSSE), precision, and accuracy of non-weighted and weighted random effects meta-analysis model with two-step Dersimonian-Laird (DSLR2) estimate applied in simulated data. Figure S1. Number of differentially expressed genes and minimum sum of squared errors of Dersimonian-Laird (DSL), two-step (DSLR2)‚ and Bayesian random-effects (BRE) models with different lengths of uniform priors for between-study variance estimation in simulated data. Table S3. Performance of Bayesian random-effects models by different levels of sample sizes (some results from homogenous simulated datasets). Table S4. Performance of classical and Bayesian random-effects models by different numbers of genes (some results from H1 heterogeneous simulated datasets). Table S5. Performance of weighted random-effects models applied with two levels of sample-quality weights (some simulation results). Figure S2. Heatmaps of expression patterns of 446 differentially expressed genes in white matter in Alzheimer’s and control samples. The DE genes were detected across the three Bayesian meta-analysis models as shown in metadata D in Fig. 6. Table S6. List of 213 significantly differentially expressed genes in Alzheimer’s gene expression dataset. The DE genes detected across the DSLR2 wP6 weighted and DSLR2 and DSL unweighted models as shown in metadata C in Fig. 5. Table S7. List of 446 significantly differentially expressed genes in Alzheimer’s gene expression datasets. The DE genes detected across three Bayesian random-effect models (Models 1, 3, and 6) as shown in metadata D in Fig. 6. (PDF 886 kb) [file 12859_2018_2491_MOESM1_ESM.pdf]

Classical and Bayesian Random-Effects Meta-Analysis Models with Sample Quality Weights in Gene Expression Studies

Uma Siangphoe[1*], Kellie J. Archer[2], Nitai D. Mukhopadhyay[3]
[1]Office of Biostatistics, Center for Drug Evaluation and Research, U.S. Food and Drug Administration, Maryland. USA, [2]Division of Biostatistics, College of Public Health, The Ohio State University, Columbus, Ohio, USA. [3]Department of Biostatistics, Virginia Commonwealth University, Richmond, Virginia, USA.

Hypothesis settings in gene expression meta-analysis

Analyses in microarray studies have emphasized identifying DE genes or genes that distinguish groups of samples. Hypothesis settings in gene expression meta-analysis can be classified in three complementary hypotheses [1-3]. The first hypothesis (HSA) is framed to detect differentially expressed (DE) genes that have non-zero effect sizes in all studies

$$H_0 : \bigcap_{i=1}^{k} \{\theta_{ig} = 0\} \text{ versus } H_A : \bigcap_{i=1}^{k} \{\theta_{ig} \neq 0\},$$

where θ_{ig} represents the effect size of gene *g* in *i*th study; $i = 1, \ldots, k$. The second hypothesis (HSB) is framed to detect DE genes that have non-zero effect sizes in one or more studies

$$H_0 : \bigcap_{i=1}^{k} \{\theta_{ig} = 0\} \text{ versus } H_A : \bigcup_{i=1}^{k} \{\theta_{ig} \neq 0\}.$$

The third hypothesis (HSC) is framed for detecting DE genes with non-zero effect sizes in a majority of combined studies (>100*p* % of combined studies; $0 < p \leq 1.0$),

$$H_0 : \sum_{i=1}^{k} I\{\theta_{ig} \neq 0\} < r \text{ versus } H_A : \sum_{i=1}^{k} I\{\theta_{ig} \neq 0\} \geq r \; ; \quad r \geq pk ,$$

where $I(.)$ is an indicator function having the value 1 for $\theta_{ig} \neq 0$ and the value 0 for $\theta_{ig} = 0$.

Reference

1. Song C, Tseng GC. Hypothesis setting and order statistic for robust genomic meta-analysis. The annals of applied statistics. 2014;8(2):777.
2. Chang LC, Lin HM, Sibille E, Tseng GC. Meta-analysis methods for combining multiple expression profiles: comparisons, statistical characterization and an application guideline. BMC Bioinformatics. 2013;14:368,2105-14-368.
3. Siangphoe U, Archer KJ. Estimation of random effects and identifying heterogeneous genes in meta-analysis of gene expression studies. Brief Bioinform. 2017;18(4):602-618.

WinBUGS codes

WinBUGS codes are shown for random-effects meta-analysis of estimates y and variance v for k number of studies and qw denoted sample-quality weight.

BRE models 1:

```
model {
for(i in 1:k) {
w[i] <- 1/v[i]
y[i] ~ dnorm(theta[i],w[i])
theta[i] ~ dnorm(mu,prec)
    }
    mu ~ dnorm(0.0,0.001)
    tau ~ dunif(0,1)
    prec <- 1/(tau*tau)
    tau2 <- tau*tau
}
```

BRE models 2 and 5:

```
model {
    for(i in 1:k) {
      w[i] <- 1/v[i]
      y[i] ~ dnorm(theta[i],w[i])
      theta[i] ~ dnorm(mu*qw[i],prec)
    }
    mu ~ dnorm(0.0,0.001)
    tau ~ dunif(0,1)
    prec <- 1/(tau*tau)
    tau2 <- tau*tau
}'
```

BRE models 3 and 6:

```
model {
    for(i in 1:k) {
      w[i] <- 1/v[i]
      y[i] ~ dnorm(theta[i],w[i])
      theta[i] ~ dnorm(mu,prec*qw[i])
    }
    mu ~ dnorm(0.0,0.001)
    tau ~ dunif(0,1)
    prec <- 1/(tau*tau)
    tau2 <- tau*tau
}'
```

Supplemental Tables and Figures

These information are used for Table S1-S5 and Figure S1 in this supplemental material. N, G, K, and H denote the number of samples, the number of genes, the number of studies, the number of studies containing heterogeneous genes, respectively, all of which varied in different simulations. H0, H1, H2, and H3 are the number of {0, 1, 2, and 3} studies containing heterogeneous genes. H0 represents homogenous data. The number of truly differentially expressed genes in the simulated data were 120 genes under the HSC hypothesis testing for the simulations on 2,000 genes.

Table S1. Number of differentially expressed (DE), minimum sum of squared errors (MSSE), precision, and accuracy of non-weighted and weighted random-effects models with Dersimonian-Laird (DSL) estimate applied in simulated data.

**SQW**	**No. DE genes**				**MSSE**				**Precision**				**Accuracy**			
	H0	H1	H2	H3	H0	H1	H2	H3	H0	H1	H2	H3	H0	H1	H2	H3
NW	65	74	92	124	2.91	2.91	2.91	2.91	0.95	0.95	0.90	0.79	0.97	0.97	0.98	0.98
S1	64	66	68	71	2.91	2.91	2.91	2.91	0.95	0.95	0.95	0.94	0.97	0.97	0.97	0.97
S2	11	25	36	46	4.01	3.99	3.94	3.82	0.96	0.96	0.96	0.96	0.94	0.95	0.96	0.96
S3	6	15	27	40	4.09	4.06	4.00	3.84	0.95	0.93	0.93	0.93	0.94	0.95	0.95	0.96
S4	65	66	69	71	2.91	2.91	2.91	2.91	0.95	0.96	0.95	0.95	0.97	0.97	0.97	0.97
S5	65	67	69	71	2.91	2.91	2.91	2.91	0.96	0.96	0.95	0.95	0.97	0.97	0.97	0.97
S6	65	66	69	71	2.91	2.91	2.91	2.91	0.96	0.96	0.95	0.95	0.97	0.97	0.97	0.97
P1	64	58	52	47	2.92	3.09	3.23	3.35	0.96	0.96	0.96	0.96	0.97	0.97	0.96	0.96
P2	65	67	69	71	2.91	2.91	2.91	2.91	0.96	0.96	0.95	0.95	0.97	0.97	0.97	0.97
P3	9	24	36	47	4.04	4.02	3.97	3.85	0.96	0.96	0.97	0.97	0.94	0.95	0.96	0.96
P4	4	14	27	40	4.11	4.08	4.02	3.87	0.95	0.94	0.94	0.94	0.94	0.95	0.95	0.96
P5	65	66	68	69	2.91	2.89	2.88	2.88	0.96	0.96	0.95	0.95	0.97	0.97	0.97	0.97
P6	62	62	64	65	2.98	2.99	3.00	3.02	0.95	0.96	0.96	0.96	0.97	0.97	0.97	0.97
P7	62	62	64	65	2.98	2.99	3.01	3.02	0.96	0.96	0.96	0.96	0.97	0.97	0.97	0.97
P8	65	66	69	71	2.91	2.91	2.91	2.91	0.95	0.96	0.95	0.95	0.97	0.97	0.97	0.97
P9	65	66	69	71	2.91	2.91	2.91	2.91	0.96	0.96	0.95	0.95	0.97	0.97	0.97	0.97
P10	65	67	69	71	2.91	2.91	2.91	2.91	0.96	0.96	0.95	0.95	0.97	0.97	0.97	0.97
P11	65	67	69	71	2.91	2.91	2.91	2.90	0.96	0.96	0.95	0.95	0.97	0.97	0.97	0.97
P12	65	66	68	70	2.91	2.92	2.92	2.92	0.96	0.96	0.95	0.95	0.97	0.97	0.97	0.97
P13	65	66	68	71	2.91	2.92	2.92	2.92	0.96	0.96	0.95	0.95	0.97	0.97	0.97	0.97

Note: SQW: sample quality weights, NW: non-weight model. S1-S6 are standardized ratio weights and P1-P13 are zero-to-one-weights.

Table S2. Number of differentially expressed (DE), Minimum sum of squared errors (MSSE), precision, and accuracy of non-weighted and weighted random effects meta-analysis model with two-step Dersimonian-Laird (DSLR2) estimate applied in simulated data.

SQW	No. DE genes				MSSE				Precision				Accuracy			
	H0	H1	H2	H3	H0	H1	H2	H3	H0	H1	H2	H3	H0	H1	H2	H3
NW	69	104	139	198	1.67	1.67	1.67	1.67	0.95	0.91	0.79	0.59	0.97	0.98	0.98	0.96
S1	71	77	84	92	1.21	1.16	1.14	1.20	0.96	0.95	0.93	0.91	0.97	0.97	0.98	0.98
S2	0	11	53	73	1.21	1.16	1.14	1.20	0.84	0.95	0.96	0.94	0.94	0.94	0.96	0.97
S3	0	11	53	74	1.67	1.67	1.67	1.67	0.84	0.96	0.96	0.94	0.94	0.95	0.96	0.97
S4	70	76	83	92	1.67	1.67	1.67	1.67	0.96	0.95	0.94	0.91	0.97	0.97	0.98	0.98
S5	70	76	83	91	1.67	1.67	1.67	1.67	0.96	0.95	0.94	0.91	0.97	0.97	0.98	0.98
S6	70	76	83	92	1.67	1.67	1.67	1.67	0.96	0.95	0.94	0.91	0.97	0.97	0.98	0.98
P1	69	67	66	64	1.67	1.67	1.67	1.67	0.96	0.95	0.95	0.95	0.97	0.97	0.97	0.97
P2	70	76	83	91	1.37	1.26	1.18	1.18	0.96	0.95	0.94	0.91	0.97	0.97	0.98	0.98
P3	0	16	59	77	1.73	1.53	1.36	1.29	0.89	0.96	0.96	0.94	0.94	0.95	0.97	0.97
P4	0	5	41	67	1.73	1.72	1.71	1.71	0.89	0.93	0.95	0.93	0.94	0.94	0.96	0.97
P5	69	74	81	88	1.59	1.57	1.55	1.53	0.96	0.95	0.94	0.92	0.97	0.97	0.98	0.98
P6	66	72	78	85	1.60	1.57	1.55	1.52	0.96	0.95	0.94	0.92	0.97	0.97	0.97	0.98
P7	66	72	78	85	1.67	1.67	1.67	1.67	0.96	0.95	0.94	0.92	0.97	0.97	0.97	0.98
P8	70	76	83	91	1.67	1.67	1.67	1.67	0.96	0.95	0.94	0.91	0.97	0.97	0.98	0.98
P9	70	76	83	91	1.67	1.67	1.67	1.67	0.96	0.95	0.94	0.91	0.97	0.97	0.98	0.98
P10	70	76	83	91	1.67	1.69	1.70	1.70	0.96	0.95	0.94	0.91	0.97	0.97	0.98	0.98
P11	70	76	82	90	1.67	1.67	1.66	1.66	0.96	0.95	0.94	0.91	0.97	0.97	0.98	0.98
P12	70	76	82	90	1.67	1.67	1.66	1.66	0.96	0.95	0.94	0.91	0.97	0.97	0.98	0.98
P13	70	76	82	90	1.70	1.70	1.69	1.69	0.96	0.95	0.94	0.91	0.97	0.97	0.98	0.98

Note: SQW: sample quality weights, NW: non-weight model. S1-S6 are standardized ratio weights and P1-P13 are zero-to-one-weights.

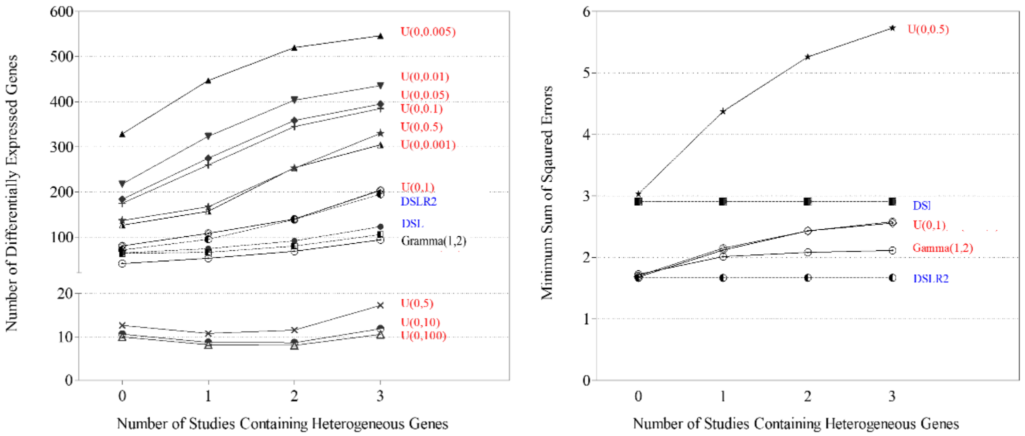

Figure S1. Number of differentially expressed genes and minimum sum of squared errors of Dersimonian-Laird (DSL), two-step (DSLR2) and Bayesian random-effects (BRE) models with different lengths of uniform priors for between-study variance estimation in simulated data.

Table S3. Performance of Bayesian random-effects models by different levels of sample sizes (some results from homogenous simulated datasets).

**Model**	**Prior**	**Sample Sizes**						
		**10**	**20**	**60**	**100**	**140**	**180**	**220**
					**No. DE genes**			
BRE	U(0,0.01)	170	267	222	218	211	210	210
BRE	U(0,0.1)	79	126	168	181	188	191	193
BRE	U(0,1)	36	63	77	80	82	83	83
BRE	U(0,5)	1	9	12	13	13	13	13
BRE	G(1,2)	29	45	46	41	47	47	47
					**MSSE**			
BRE	U(0,0.01)	4.44	5.13	7.84	10.50	13.15	15.77	18.46
BRE	U(0,0.1)	4.25	4.99	7.39	9.35	11.01	12.47	13.83
BRE	U(0,1)	2.60	2.23	1.81	1.70	1.64	1.61	1.59
BRE	U(0,5)	1.21	1.15	1.10	1.07	1.08	1.08	1.07
BRE	G(1,2)	2.63	2.35	1.99	1.73	1.78	1.73	1.70
					**Precision**			
BRE	U(0,0.01)	0.51	0.44	0.54	0.55	0.57	0.57	0.57
BRE	U(0,0.1)	0.95	0.87	0.71	0.66	0.64	0.63	0.62
BRE	U(0,1)	1.00	1.00	1.00	1.00	1.00	1.00	1.00
BRE	U(0,5)	1.00	1.00	1.00	1.00	1.00	1.00	1.00
BRE	G(1,2)	1.00	1.00	1.00	1.00	1.00	1.00	1.00
					**Accuracy**			
BRE	U(0,0.01)	0.94	0.92	0.95	0.95	0.95	0.96	0.96
BRE	U(0,0.1)	0.98	0.99	0.98	0.97	0.97	0.96	0.96
BRE	U(0,1)	0.96	0.97	0.98	0.98	0.98	0.98	0.98
BRE	U(0,5)	0.94	0.94	0.95	0.95	0.95	0.95	0.95
BRE	G(1,2)	0.95	0.96	0.96	0.96	0.96	0.96	0.96
					**AUC**			
BRE	U(0,0.01)	0.83	0.95	0.97	0.97	0.98	0.98	0.98
BRE	U(0,0.1)	0.81	0.95	0.99	0.98	0.98	0.98	0.98
BRE	U(0,1)	0.65	0.76	0.82	0.84	0.84	0.84	0.85
BRE	U(0,5)	0.50	0.54	0.55	0.55	0.55	0.55	0.55
BRE	G(1,2)	0.62	0.69	0.69	0.67	0.69	0.70	0.69

Note: DE: differentially expressed, MSSE: minimum sum of squared error, AUC: area-under ROC curve, BRE: Bayesian random-effects model, U: uniform, and G: gamma.

When varying sample sizes, the Bayesian RE models identified less genes for sample sizes <60. The DE gene detection and the MSSE were stable for sample sizes >60. The BRE with a uniform(0,1) prior had consistently high precisions and was able to maintain high overall accuracies for all sample sizes >60. As anticipated, these findings were similar to the results found in the classical RE models (Siangphoe U, el al. 2017).

Table S4. Performance of classical and Bayesian random-effects models by different numbers of genes (some results from H1 heterogeneous simulated datasets).

Model	Prior	No. of Genes	No. of True DE genes	No. of DE Genes	MSSE	Precision	Accuracy	AUC
DSL	-	10,000	600	329	2.9	0.96	0.97	0.99
DSLR2	-	10,000	600	379	1.7	0.95	0.97	0.96
BRE	U(0,1)	2,000	120	108	2.2	0.94	0.99	0.92
BRE	U(0,1)	10,000	600	360	2.4	0.98	0.97	0.80

Note: DE: differentially expressed, MSSE: minimum sum of squared error, AUC: area-under ROC curve, DSL: DerSimonian-Laird model, DSLR2: two-step estimate of DerSimonian-Laird model, BRE: Bayesian random-effects model, and U: uniform.

It is noteworthy that when the number of genes in the analyses increased, the classical RE models performed stably. In contrast, the overall accuracy by AUC in the BRE model with a uniform(0,1) prior was reduced.

Table S5. Performance of weighted random-effects models applied with two levels of sample-quality weights (some simulation results).

Models	Levels of sample quality weights in simulated data	No. DE Genes				MSSE				Precision				Accuracy				AUC			
		H0	H1	H2	H3	H0	H1	H2	H3	H0	H1	H2	H3	H0	H1	H2	H3	H0	H1	H2	H3
DSLR2 w_{P6}	Beta (10,1)	66	72	78	85	1.6	1.6	1.6	1.6	0.96	0.95	0.94	0.92	0.97	0.97	0.97	0.98	0.76	0.78	0.80	0.82
DSLR2 w_{P6}	Beta (5,1)	61	64	68	73	1.6	1.6	1.6	1.5	0.96	0.96	0.95	0.94	0.97	0.97	0.97	0.97	0.74	0.75	0.77	0.78

Note: $\overline{w}_{P6}$ is an average of w_{P6}, $w_{P6} = \left(\sigma_{ig}^{2(w_{P1})} + \hat{\tau}_g^2\right)^{-1}$ over the total samples; $w_{P1} \in \left\{2^{-S_{ij}}, 0.01\tilde{P}_{ij}\right\}$, $\tilde{P}_{ij}$ denoted percent of present calls, S_{ij} denoted standardized quality indicators of the *j*th sample in the *i*th study. DE: differentially expressed, MSSE: minimum sum of squared error, AUC: area-under ROC curve, DSLR2: two-step estimate of Dersimonian-Laird model.

The sample-quality weights assumed to follow beta $(\alpha = 10, \beta = 1)$ for the zero-to-one weights presents as higher quality weights than the weights assumed with beta $(\alpha = 5, \beta = 1)$. Apparently, the samples with higher quality weights detected more DE genes and had higher overall accuracy by AUC than the samples with lower quality weights.

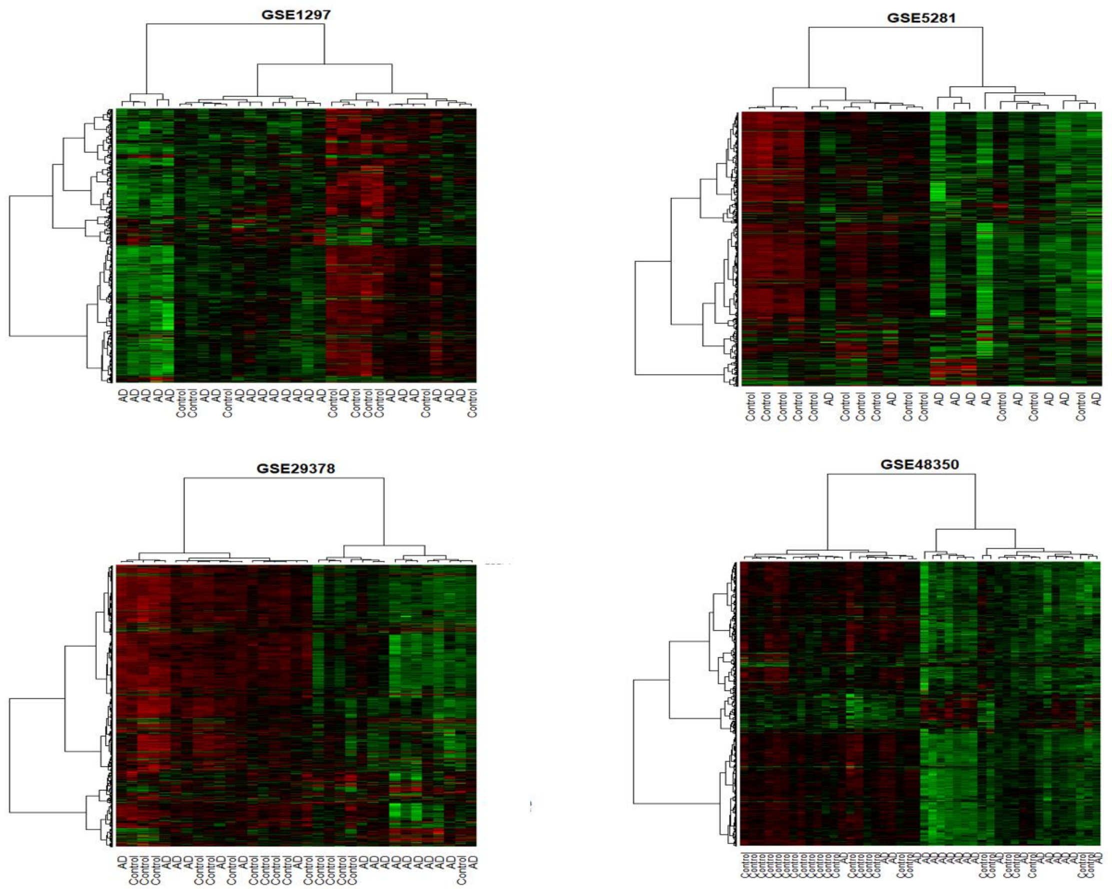

Figure S2. Heatmaps of expression patterns of 446 differentially expressed genes in white matter in Alzheimer’s and control samples. The differentially expressed genes were detected across the three Bayesian meta-analysis models as shown in metadata D in Figure 6.

Table S6. List of 213 significantly differentially expressed genes in Alzheimer's gene expression dataset. The differentially expressed genes detected across the DSLR2 w_{P6} weighted and DSLR2 and DSL unweighted models as shown in metadata C in Figure 5.

AACS, ABCA1, ABI3BP, ACTL7B, ADCY8, AHCYL1, AHNAK, AKR1C3, AMOT, ANG, ANGPT1, AP3B2, APLNR, ARHGDIG, ASAP3, ATP13A2, ATP6V0E1, BBX, BCL11A, BSN, C22orf31, CAMK2N1, CAMTA1, CDC42EP4, CEP112, CFI, CHGA, CLIP3, CNR1, COLEC12, COX4I1, COX6A1, CRABP1, CRLF1, CRTAP, CS, CUL2, DDAH2, DDR2, DHRS3, DLEC1, DLGAP2, DNALI1, DPYSL3, DPYSL4, DRAM1, ECM2, ELOVL4, ENTPD2, EPB41L1, ERBB2, ERBB2IP, EXPH5, EZR, FAM107A, FAM174B, FGF12, FIBP, FKBP11, FLNC, FMO2, FOXJ1, FOXO1, FSD1, FXYD7, FYN, GABRG2, GAD1, GAD2, GALNT10, GAS7, GFAP, GJA1, GMPR, GNA12, GNAI2, GNG3, GPI, GRIN1, GYPC, HERC1, HLA-DPB1, HMGCR, HSPA12A, ID4, IL13RA1, IMP4, INA, IQCG, IQCK, ITGB4, ITGB5, ITPKB, ITSN1, KANK1, KCNQ2, KIF21B, KITLG, LCAT, LIFR, LIMS1, LPAR4, LPCAT4, LPIN1, LPP, LTBP1, LTF, MAP1A, MAST1, MAST3, MCF2, MFAP4, MLC1, MRPL35, MRPS12, NDUFA10, NEBL NEFL, NPAS3, NRGN, NSG1, NXT2, P4HTM, PABPC1, PAX6, PBXIP1, PLCD1, PLCE1, PLEKHA4, PLSCR4, PMP2, POLB, POLRMT, PPFIA1, PPFIA4, PPP1R13L, PRKCZ, PRPF40A, PSD4, PSTPIP1, PTTG1IP, PUS7, RAB13, RAB26, RAP1GDS1, RARRES3, RASL12, RBL2, RCAN2, RFX4, RGS4, RIMS2, RND2, RNF123, RNF19A, RNF41, RNFT2, RPGR, RPP40, RPRM, RPS6KC1, RUSC1, S100A2, SCG5, SEC11A, SEL1L3, SEPT6, SERPINA5, SERPING1, SERPINI2, SHROOM2, SLC12A5, SLC14A1, SLC25A11, SLC25A12, SLC27A6, SMAD5, SMOX, SNX5, SORBS1, SRPX2, SS18L1, SSFA2, SSPN, SST, STAT3, STMN2, SUGP2, SV2A, SWAP70, SYF2, SYNC, SYP, TAC1, TBL1X, TCEA2, TCF7L2, TJP2, TMEM151B, TMEM160, TNPO1, TUBG2, UCHL3, VAT1, VCAN, VSNL1, WIF1, ZFAND3, ZFP69B, ZHX3, ZMYND10, ZNF217, ZNF460

Table S7. List of 446 significantly differentially expressed genes in Alzheimer's gene expression datasets. The differentially expressed genes detected across three Bayesian random-effect models (Models 1, 3, and 6) as shown in metadata D in Figure 6

AACS, AASDHPPT, ABCA1, ACLY, ACOT7, ADAM22, ADAM23, ADARB1,AFF2, AGK, AMPH, ANGPT1, ANP32C, AP2S1, AP3B2, AP3D1, AP3M2, APBA2, APMAP, ARFGEF1, ARHGDIG, ARHGEF9, ARPC5L, ASIC2, ASNS, ASPHD1, ATAT1, ATP1A1, ATP1A3, ATP2A2, ATP2B2, ATP5B, ATP5C1, ATP5D, ATP5G1, ATP5H, ATP5L, ATP6AP1, ATP6V0B, ATP6V0E1, ATP6V1B2, ATP6V1E1, ATP6V1G2, ATP8A2, ATPIF1, ATR, ATRN, ATRNL1, ATXN7L3B, BCL2, BEX1, BEX4, BPGM, BSN, C10orf88, C12orf10, C14orf2, C16orf45, C1orf216, C2CD5, C2orf47, C5orf22, CA10, CABYR, CACNA2D3, CADPS, CALY, CAMK1, CAMK2N1, CAMKV, CAPRIN2, CCK, CDC40, CDC42EP4, CDK5, CDKN2D, CGREF1, CHGB, CHN1, CISD1, CLIP3, CLTA, CNR1, COPS3, COPS7A, COPZ2, COQ6, COX4I1, COX6C, CP, CREBBP, CRYM, CS, CUL2, CYCS, CYP4F12, DAP3, DCTN1, DDX41, DEAF1, DGUOK, DHRS11, DHRS3, DIRAS3, DLEC1, DLG2, DLGAP2, DMXL2, DNASE2, DNM1, DNM1L, DNM3, DOCK3, DOPEY1, DROSHA, DYNC1H1, DYNC1I1, ECM2, EEF1A2, EGFR, EHD3, ELF1, ELOVL4, ELOVL6, ENC1, ENO2, ENTPD2, ENTPD3, EPB41L1, EPS15, ERC2, FAM111A, FAM127A, FAM162A, FAM174B, FAM188A, FAM216A, FAM60A, FAM98A, FAR2, FGF12, FH, FHL2, FIBP, FKBP3, FMO2, FOCAD, FOXJ1, FOXO1, FRMPD4, FSD1, FXN, FYCO1, FYN, GABBR2, GABRG2, GAD, GAD2, GCC2, GLS2, GNAI2, GNG3, GNG4,GOT1, GPHN,GPI, GPRASP1, GRIA2, GRIN1, GRM1, GSTA4, GUCY1B3, GUK1v GYPC, HAGH, HARS, HERC1, HMGCR, HMP19, HN1, HNRNPUL1, HPRT1, HSPA12A, IGF1R, IMMT, IMP3, IMP4, INA, INPP5F, ITPKB, ITSN1, KAT6A, KCNN3, KCNQ2, KIAA0513, KIAA1324, KIF21B, KIFAP3, LARGE, LCMT1, LDB2, LEMD3, LGALS8, LPAR4, LPCAT4, LPIN1, LPP, LRPPRC, LRRC8B, LY6H, MAK16, MAP1A, MAP2K1, MAP2K4, MAP3K9, MAPK9, MAST3, MCF2, MCTS1, MDH1, MDH2, MICU1,MKKS, MLLT11, MOAP1, MPP1, MPPED2, MRPL15, MRPL17, MRPL35, MRPS11, MRPS17, MRPS22, MTMR11, MTSS1L, MTX2, MXI1, MYL12B, MYT1L, NAP1L2, NAP1L3, NCALD, NDN, NDRG3, NDRG4, NDUFA10, NDUFA3, NDUFA4, NDUFA8, NDUFA9, NDUFS3, NDUFS5, NDUFV2, NECAP1, NEDD8, NEFL, NEFM, NELL1, NETO2, NFIB, NIPSNAP3B, NLK, NME1, NMNAT2, NOVA1, NREP, NRGN, NRIP3, NRN1, NSF, NSG1, NUPL2, OGDHL, OPA1, ORC5, P4HTM, PAGE1, PAX6, PDCD1LG2, PEX11B, PIN1, PLCD1, PLCE1, PLCL2, PLD3, PLEC, PLEKHA4, PLK2, PLSCR4, PLXNB2, PMFBP1, PNMAL1, PNO1, PODXL2, POLB, POLRMT, POP7, PPFIA4, PPIA, PPIP5K1, PPM1H, PPME1, PPP1R13L, PPP2CA, PPP3CB, PREP, PREPL, PRKCZ, PRMT1, PRPF40A, PSD4, PSMD8, PTDSS1, PTGES2, PTPRE, PTPRR, PTRH2, PTS, PVRL3, RAB11A, RAB26, RAB27A, RAB2A, RAB6A, RAD51C, RAP1GDS1, RARS, RBFOX2, RGS17, RGS7, RHOQ, RIMBP2, RIT2, RND2, RNF123, RNF41, RNFT2, RNMT, RNPS1, RPH3A, RPP40, RPS6KC1, RUNDC3B, RWDD2A, RXRA, SCAMP2, SCG5, SCN2A, SCN3B, SDHA, SEC22A, SEC61A2, SEH1L, SEPT6, SERPINI2, SEZ6L2, SLC12A5, SLC25A11, SLC25A12, SLC25A14, SLC25A4, SLC4A1AP, SLIRP, SLITRK3, SMARCA4, SMO, SMOX, SMYD2, SNAP25, SNAP91, SNCB, SOX2, SPAG7, SPIN2A, SPINT2, SRM, SRPR, SS18L1, SSPN, STAU2, STMN2, STX6, STXBP1, SULT4A1, SUSD4, SV2B, SYDE1, SYN1, SYN2, SYNGR1, SYNJ1, SYT1, TAGLN3, TAZ, TBC1D31, TBC1D9, TBCC, TBCE, TBL1X, TBPL1, TCEA2, TCF7L2, TERF2IP, TGFBR3, THOC5, TMEM151B, TMEM160, TMEM246, TMEM59L, TMEM70, TMEM97, TNPO1, TOMM34, TOMM70A, TOR1A, TPD52, TPI1, TRAP1, TRAPPC2, TRIM37, TRIM9, TRIOBP, TSPAN13, TSPAN7, TSSC1, TUBA1B, TUBA4A, TUBB3, TUBG1, TUBG2, TXNDC9, UBE2M, UBE2S, UCHL1, UCHL3, UQCC1, UTP11L, VSNL1, WDR47, WDR7, WFDC1, XK, YWHAH, ZFP36L1, ZNF365, ZNHIT3
